# Supplementary material for: Generation of 2-mode scale-free graphs for link-level internet topology modeling
Source: PLoS One. 2020 Nov 9;15(11):e0240100. doi: 10.1371/journal.pone.0240100 (PMC7652253; doi:10.1371/journal.pone.0240100)
Supplement: S1 File — (ZIP) [file pone.0240100.s001.zip › SubNetG_ revision/subnetG_final.pdf]

Generation of 2-mode Scale-Free Graphs for link-level Internet Topology Modeling

Khalid Bakhshaliyev[1], Mehmet Hadi Gunes[2]

1 Department of Computer Science and Engineering, University of Nevada, Reno
2 School of Systems and Enterprises, Stevens Institute of Technology

Abstract

Comprehensive analysis that aims to understand the topology of real-world networks and the development of algorithms that replicate their characteristics has been significant research issues. Although the accuracy of newly developed network protocols or algorithms does not depend on the underlying topology, the performance generally depends on the topology. As a result, network practitioners have concentrated on generating representative synthetic topologies and utilize them to investigate the performance of their design in simulation or emulation environments. Network generators typically represent the Internet topology as a graph composed of point-to-point links. In this study, we discuss the implications of multi-access links on the synthetic network generation and modeling of the networks as bi-partite graphs to represent both subnetworks and routers. We then analyze the characteristics of sampled Internet topology data sets from backbone Autonomous Systems (AS) and observe that in addition to the commonly recognized power-law node degree distribution, the subnetwork size and the router interface distributions often exhibit power-law characteristics. We introduce a SubNetwork Generator (SubNetG) topology generation approach that incorporates the observed measurements to produce bipartite network topologies. In particular, generated topologies capture the 2-mode relation between the layer-2 (i.e., the subnetwork and interface distributions) and the layer-3 (i.e., the degree distribution) that is missing from the current network generators that produce 1-mode graphs. The SubNetG source code and experimental data is available at `https://github.com/netml/sonet`.

Keywords: Autonomous Systems; Internet backbone; Network topology; Power-law

1 Introduction

As the largest human-made complex network, the Internet grows with no central authority. Internet connectivity is provided by tens of thousands of Autonomous Systems (AS), organizations that maintain a physical network, or a group of networks. Each AS is assigned a unique identification number to be employed with the Border Gateway Protocol (BGP), and possesses Internet Protocol (IP) address ranges to be utilized for unique identification of devices across the Internet and routing of the network traffic. A large number of decentralized AS, which vary in size and geographic footprint, connect individuals, businesses, and organizations. Each AS produces its network based on it's own economic and technical objectives [1]. Overall, the Internet evolves with an interplay between *cooperation* so that the network works efficiently, and

competition so that network providers earn a profit. Knowledge of the underlying network graph helps in understanding the large scale characteristics and dynamics of the Internet [2]. Network practitioners test new protocols and systems using simulations or emulations, but more realistic results can be obtained when employed topologies reflect the characteristics of genuine networks [3–6]. If the synthetic topology used during the simulation does not reflect the crucial characteristics of real networks, evaluation results will be misleading and the expected performance will not be observed when the system is deployed in the wild [7, 8]. Hence, network generators are needed to produce synthetic networks that reflect the underlying properties of genuine networks.

Modeling of interactions and generation of representative networks has been a significant challenge in various research fields such as modelling an efficient network for power-grids [9] and water distribution networks [10], and several generation models have been developed [11–14]. Internet topology modeling focuses on understanding local and global characteristics of the Internet backbone, and construction of graph models that mimic the observed topological features. When one samples a network to generate synthetic networks, many of the underlying relations may get omitted or altered. This may result in a graph that does not resemble the original network for crucial characteristics [15]. For example, when designing algorithms to find communities of multilingual users in social networks, language metric needs to be taken into account along with the traditional network connectivity [16]. Likewise, when modeling protein interactions to detect proteins dedicated to a specific cellular process, one needs to consider the neighborhood expression variance of proteins [17].

Network topology generation involves producing synthetic graphs that replicate certain characteristics of the original network. The randomness of the generated topology depends on the set of metrics that are targeted. As the number of constraints increases, the network is described in greater detail, and hence the generated topology better resembles the reference graph. In the utmost case, one can define a complete set of metrics that uniquely describe every aspect of a network, and the generated topology will be isomorphic to the reference. However, increasing the number of constraints raises the algorithmic complexity to find a graph meeting all constraints. Hence, topology generators need to balance between the complexity and the representativeness.

The Internet traffic between two systems is transferred through a set of routers (i.e., layer 3 of the Internet protocol suite) interconnected via various link technologies (i.e., layer 2 of the Internet). A router might be connected via a direct link (i.e., a point-to-point link) or through a multi-access medium (e.g., bus, switch, Fiber Distributed Data Interface (FDDI) ring, etc.) using a single network interface. Routers have multiple network interfaces and each interface has a dedicated IP address. A group of IP addresses is typically represented as a subnet where the most significant bits are the same. When routers are connected over a link, the connected interfaces are assigned IPs from the same subnet range, and hence are referred to as a subnetwork. Note that, we refer to the logical IP address range as subnet, and the physical connectivity among a group of network interfaces as a subnetwork.

Multiple nodes are connected through a multi-access link, and they are commonly deployed in the Internet backbone [18, 19]. Previous synthetic Internet topology generators have often ignored multi-access links, the building block of the networks. In a topological perspective, multi-access links have two major implications, namely, pairwise one-hop connectivity between subnetwork devices and sharing of the link bandwidth. In generating link-level synthetic Internet topologies, we should consider interface and subnetwork distributions to reflect the multi-access links [20] in addition to the observed degree distribution [21]. The degree of a node is typically defined as the number of nodes it is connected and is typically higher than the number of network interfaces of the device. A 2-mode graph representation reflects the actual interfaces of

a node as well as it's one-hop connectivity via subnetworks. Modeling of Internet topology as a 2-mode graph helps us capture the building blocks of the networks along with the large-scale characteristics of the Internet backbone.

As we are interested in the link-level Internet topology, we focus on the *interface distribution* and *subnetwork distribution* as two metrics to model the underlying connectivity in addition to the commonly utilized *degree distribution.* Interface distribution reflects the number of network interfaces of devices (such as routers, servers, etc) and plots the number of devices with a given number of interface count. Likewise, subnetwork distribution exhibits the number of network interfaces connected to subnetworks (i.e., point-to-point links or multi-access links) and plots the number of subnetworks with a given number of attached devices. The degree distribution ignores the access medium over which a single interface connects multiple nodes over a subnetwork. A link-layer device (e.g., a bus, switch, or FDDI ring) or a link-layer network (e.g., a switch forms the single collision domain of the subnetwork. Note that "degree" indicates the number of one-hop neighbors , and is proportional to the number of interfaces and the size of the subnetworks those interfaces are attached.

In order to produce realistic synthetic topologies, we analyzed the backbone Internet topologies sampled by the Autonomous System Mapper (ASM) [22]. Our analysis of several AS reveals that many have power-law distribution patterns in the degree, subnetwork, and interface distributions [20]. In this study, we derive the power-law exponent of the degree, subnetwork and interface distributions. We also derive the condition for the power-law exponent ranges that ensure the existence of a connected network when interface and subnetwork distributions are power-laws. We then utilize these results in the SubNetwork Generator (SubNetG) topology generator.

Focusing on the relation between layer 2 (i.e., subnetwork and interface) and layer 3 (i.e., degree) in the Internet, this study aims to provide a link-level network topology generation mechanism focusing on the building blocks of communication networks. Consideration of the multi-access links in the synthetic topology generation addresses a missing level of granularity in the Internet topology models. Additionally, modeling multi-access links based on genuine Internet measurements produces synthetic topologies that better capture the underlying characteristics of the backbone networks.

In the rest of the paper, Section 2 summarizes synthetic network generation approaches. Section 3 summarizes the missing component of the current Internet topology generators. Section 4 introduces the SubNetwork Generator (SubNetG) that produces 2-mode graphs where the distributions are power-law. Section 5 presents evaluations of SubNetG, and Section 6 concludes the paper.

2 Related Work

In this section, we present an overview of synthetic network generation approaches that could be employed to represent Internet topologies.

Random Network Models: Initial network generation relied on traditional random network frameworks such as the *Erdos−Renyi* model [13] where nodes are randomly interconnected. Random network model is not a good representation of the Internet topology due to its failure to capture many crucial properties such as the heavy-tailed degree distribution and high clustering.

Hierarchical Network Models: Hierarchical topology generators mimic network deployment practices. *Tiers* [23] captures the hierarchical aspect of the Internet by implementing the network hierarchy where nodes are linked with a minimum spanning tree at LAN and MAN levels. Similarly, *GT-ITM* [24] generates hierarchical networks by building transit and stub domains. GT-ITM generates a connected random graph in which each node is considered as a transit domain and then grows each domain to

contain a random graph. After expanding the operation for n-levels, a number of random graphs are generated and connected to each node as stubs. Finally, *IGEN* [25] implements Internet engineering heuristics to populate networks based on design choices. While focusing on the network growth processes, hierarchical network generators miss large-scale characteristics of the Internet.

Small-World Network Models: Many real-world networks, including the Internet, have shown to exhibit the small world characteristic, i.e. high clustering and low characteristic path length. *Watts−Strogatz* model interpolates ordered lattices with large clustering coefficients and purely random networks with small average path lengths to produce small world networks [14]. Although small world networks obtain high clustering and low average path length, they lack degree characteristics observed in the Internet topologies.

Scale-Free Network Models: Internet topologies are shown to exhibit power-law degree distribution at AS level and router level [21, 26]. These studies shifted the attention to *degree-based* generators [27]. In order to bridge the gap between the local and global properties of the Internet, statistical physics-based approaches were proposed [28]. *Preferential attachment* mimics network growth where edges are not placed randomly but have a tendency to connect to high degree nodes [29]. The Boston university Representative Internet Topology gEnerator (*BRITE*) [6] generates networks with a power-law degree distribution and allows locality-based preferential attachment to generate hierarchical networks. BRITE also utilizes the Erdos-Renyi model where the probability of the existence of a link between two nodes is inversely proportional to the distance between the nodes. *Inet* [30] produces synthetic Internet graphs that have power-law degree distributions. *Jellyfish* [31] uses a core formed around central nodes to obtain topologies that have core-periphery structures.

dK-Series Network Models: *dK-series* provides a basis to characterize a graph [32]. For an n node network 0K-graph only matches the average degree, 1K-graph matches the degree distribution, 2K-graph matches the Joint Degree Distribution, and so on. The nK-graph is isomorphic of the original graph. Researchers have introduced a methodology for the rescaling process to produce different sized graphs with the same 2K-series characteristics [33], but there is no known efficient generation mechanism for higher dK matches.

Dual Internet topology generator: Center for Applied Internet Data Analysis (CAIDA) introduced a scalable tool that generates dual Internet topologies that aim to capture both router-level and AS-level network characteristics [34]. They use the methods from [35] to generate a network of ASes and methods from [36] to generate a router network for each AS. Nonetheless, the generated networks do not reflect the 2-mode link-level characteristics.

3 Link-Level Internet Characteristics

Researchers have studied various graph metrics to summarize reference graphs. Although many of these metrics (such as degree distribution, clustering, and characteristic path length) are essential to specify a network, there may be metrics that are better suited for a specific domain. Current topology generators do not consider multi-access links and model all subnetworks as point-to-point links. This may become an important hurdle for achieving accurate network simulations/emulations. In this study, in addition to the commonly utilized degree distribution, we focus on the interface and subnetwork distributions to capture the link-level connectivity of the Internet backbone networks. Subnetworks provide link-level connectivity among a set of device interfaces.

Before examining the interface and subnetwork distributions, we first illustrate these

Fig 1. Sample Topologies

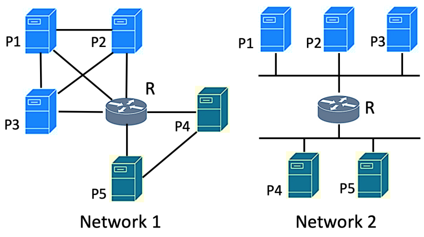

distributions on a toy network. Figure 1 shows two different topologies with the same node size. Network 1 is composed of only point-to-point links, whereas Network 2 involves multi-access links that enable one-hop connectivity to multiple nodes through a single link. Nodes belonging to the same subnetworks are marked with the same colors. In both topologies, nodes have the same node degrees, and hence the degree distributions are the same. However, when the number of interfaces is compared, the distinction of multi-access links becomes clear. Although router R has the same degree of 5 in both of networks, its interface count is 5 in Network 1 but 2 in Network 2. While both networks have the same degree distribution, their interface distributions are considerably different as shown in Figure 2.

To show the difference of the network representations in practice, we perform a maximum throughput simulation based on the Internet2 [37] topology, backbone of the academic networks in the U.S. Network throughput is an important metric to measure the maximum amount of data that could be sent from a source to a destination. Gathering the ground truth information from configuration files of Internet2 routers [38], we found that Internet2 backbone had 440 nodes (i.e., routers and servers) connected over 90 subnetworks. We observe 60% of the Internet2 subnetworks are multi-access links with more than two attached network interfaces.

We use ns-3 network simulator [39] to simulate the throughput where the Internet2 backbone is modeled as a 1-mode graph (where routers are assumed to be directly interconnected via point-to-point links) and a 2-mode graph (i.e., network interfaces are connected over subnetworks). We then assume data traffic is sent between 10 random source and destination pairs and repeat each scenario 100 times. We use the same source-destination pairs and assume a link bandwidth of 1 Gb for transferring data in both representations.

Table 1 shows the 5-number summary of cumulative throughput between randomly selected node pairs. We observe that 1-mode representation of the topology, which ignores the underlying subnetworks, produces an inflated bandwidth. The traditional 1-mode model of the internet topologies ignores the underlying multi-access links that share the communication link among multiple subnetwork interfaces and hence has a single collision domain that limits simultaneous data transfers. Hence, the 1-mode

Fig 2. Interface, Subnetwork and Degree Distribution of Sample Topologies

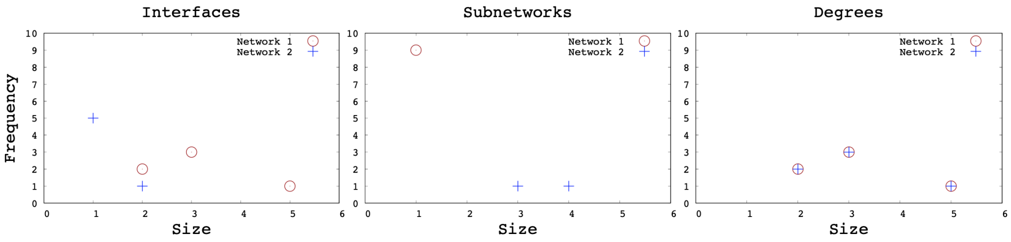

Table 1. Network throughput (Mbps) simulation on Internet2 topology

Representation	Min	1st quartile	Median	3rd quartile	Max
1-mode	2,682	2,727	2,727	2,728	2,729
2-mode	317	381	392	402	424

model produces results that are significantly higher than the achievable bandwidth that is reflected in the 2-mode model. This experiment shows the importance of the 2-mode graph modeling of the link-level Internet.

We showed the effect of neglecting multi-access links and the corresponding collision domain between the systems connected over a shared link. Overall, current topology generators that only use point-to-point links considerably overestimate the maximum throughput of networks, even if they could capture the cliquishness of the underlying topologies. As the inflation is by an order of n^2 for n interfaces, the difference in the bandwidth of the simulations will be exponentially higher for larger subnetworks.

4 SubNetG: SubNetwork Generator

In this section, we present the SubNetwork Generator (SubNetG) to produce synthetic topologies that reflect the link-level characteristics of the Internet backbone. In particular, we generate network topologies in the proximity of the desired network size, node interface distribution, and subnetwork size distribution. The generation process also converges the degree distribution to the measured power-law distribution.

4.1 Obtaining Power-Law Distribution with a Cutoff

Power-law distribution is a distribution where frequency of attributes vary as a power of the attribute, and follows the exponential form $\mathbf{F_i} = \mathbf{Ai}^{-\alpha}$ where i indicates the attribute such as degree, A is the scaling coefficient and α is the power-law exponent. In a power-law distribution, there are considerably abundant small values (i.e., F_i is high for small values of i) while extremely large values are rare but possible. As $\log(\mathbf{F_i}) = \log(\mathbf{Ai}^{-\alpha}) = \log(A) + (-\alpha)log(i)$, we observe a power-law distribution as a line in the log-scale where α exponent determines the slope of the curve. While α exponent uniquely determines the distribution, the scaling coefficient A reflects the network size.

We could generate a power-law distribution with any desired α using the *transformation method* [40], such that, one can produce a number of uniformly distributed random numbers within the $0 \leq r < 1$ range and then convert the numbers using $x = (1 - r)^{-1/(\alpha-1)}$ transformation equation. The resulting distribution would be a power-law within the range of $1 \leq x < \infty$ and the given power-law exponent α. This approach would yield a set of numbers having a distribution of the given power-law exponent. However, with this method, we can only generate interface and subnetwork distributions with a given number of interfaces, but we don't have direct control over the number of nodes.

Algorithm 1 generates a set of numbers with the specified number of nodes N and power-law exponent α. The *min* and *max* parameters are used to adjust the minimum and maximum degrees in the distribution. Routers and switches have a physical footprint, and hence having a router or switch with a very large number of interfaces is impractical. The *max* interface routers or subnetworks is determined based on extensive measurement of ASes [22]. Similarly, an optional parameter *min* is used to adjust the minimum degree nodes for subnetworks since subnetworks have at least two interfaces. If *min* is not specified, a value of 1 is used.

Algorithm 1: PowerLawCutoff(N, α, max, $[min]$)

1 $Size_i \leftarrow 0 \; \forall$ i
2 $Distribution_i \leftarrow Ai^{-\alpha} \; \forall$ i
3 $UpperBound \leftarrow \sum_i Ai^{-\alpha}$
4 **for** $n \leftarrow 1 \ldots N$ **do**
5     $R \leftarrow$ ***random***$[0, UpperBound]$
6     $i \leftarrow min$ *//default min = 1*
7   **while** $R > 0$ & $i \leq max$ **do**
8         $R \leftarrow R - Distribution_i$
9         $i{+}{+}$
10     $Size_i{+}{+}$

In Line 2 of the algorithm, a temporary distribution curve is generated with the given power-law exponent α but with a much larger distribution coefficient A. In Line 3, the integral of the temporary distribution curve is calculated. Then, the algorithm iterates number of nodes N times to obtain a skewed probability distribution using the temporary curve. Line 5, generates uniformly distributed random numbers so that the loop in Lines 7-9 determines the number of interfaces for the node. Finally, Line 10 increments the distribution count of the determined size i.

At the end of the algorithm, the sum of values in the final distribution $\sum_{\mathbf{i}} \mathbf{Ai}^{-\alpha}$ will approximate the desired network size N. Downscaling in integers introduces error due to rounding, and the sum of values might be a bit different than the intended distribution. Hence, the final network size might be different from the expected network size by a couple of nodes.

Using Algorithm 1, we generated multiple networks with various number of nodes (i.e., 1K, 10K, and 100K) and power-law exponents of 2, 2.5, and 3. Figure 3 shows the Probability Distribution Function (PDF) and Complementary Cumulative Distribution Function (CCDF) of interface distributions for networks without any cutoff using

Fig 3. Sample interface distributions with power-laws of 2, 2.5, 3

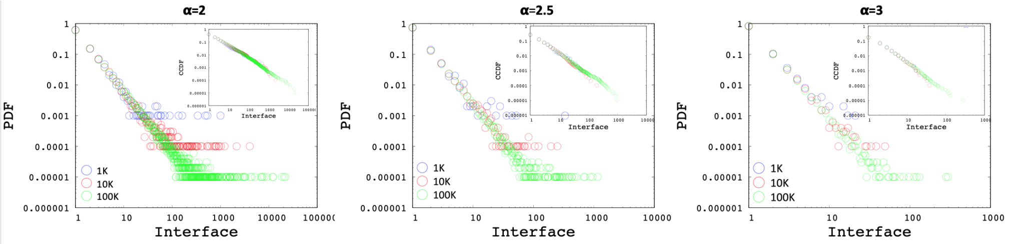

Fig 4. Sample subnetwork distributions with power-laws of 2, 2.5, 3

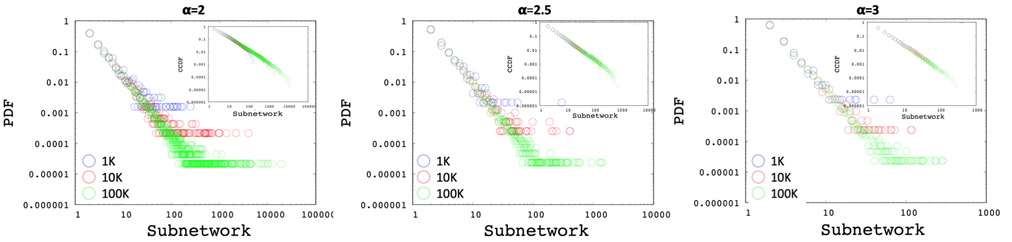

Fig 5. Change in the power law exponent with a 40% cut-off

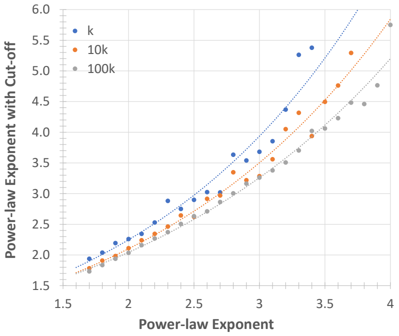

power-law exponents of 2, 2.5, and 3 respectively. Similarly, Figure 4 presents the subnetwork distribution which uses a min subnetwork size of 2 without a *max* cutoff. As observed in the figures, the generated interface and subnetwork sizes can be unrealistically high especially for low power-law exponents.

The *max* cutoff shifts the power-law distribution of the network and depends on the network size and the expected power-law exponent. To determine the produced alpha value change based on the cutoff and input alpha value, we generated 3 sets of networks with 1k, 10k and 100k nodes and a cutoff value of 40% of the network size. In each set, we run the algorithm 15 times with varying power-law exponents and measured the resulting power-law exponent due to the *max* cut-off. Figure 5 shows the median alpha values of the resulting distributions and their exponential regression.

We analyzed the largest AS networks and their interface and subnetwork sizes [22]. The maximum interface size for the largest AS networks was around 2^{12}. Similarly, the maximum subnetwork size was around 2^{15}. Hence, we picked these values as the *max* cutoff for the network generation.

4.2 2-mode Graph Generation Approach

Subnetwork based Internet topologies cannot be modeled as a conventional 1-mode graph as it requires a distinction between routers and subnetworks. A hypergraph *H=(N,S)* is a generalized graph form where N is the set of nodes and S is the set edges. Each element of S represents a subset of N, which is connected through the same subnetwork. Hypergraphs are also illustrated using 2-mode bipartite graphs. In order to maintain the subnetwork relations among the nodes, we use an undirected bipartite graph where vertices are either nodes (e.g., a router, a server, or a computing device with one or multiple IP addresses) or subnetworks, as shown in a toy network in Figure 6. Each node is attached to at least one subnetwork (shown as clouds), and each subnetwork is attached to at least two nodes (shown as routers). The number of attachments for each subnetwork and node is shown on the figure.

In order to generate a network, the number of nodes N, the power-law exponent of interface distribution $\alpha_{\mathbf{I}}$, and the power-law exponent of subnetwork distribution $\alpha_{\mathbf{S}}$ are

Fig 6. Sample bipartite graph

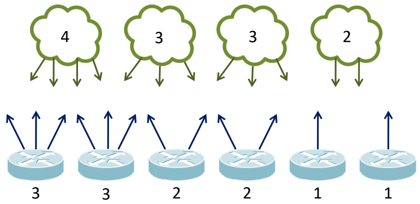

provided by the user or obtained from the measurement data. Given the slope $\alpha_{\mathbf{I}}$, the area beneath the interface distribution curve should match N (i.e., $\sum_{\mathbf{i}} \mathbf{ID_i} = N$ where ID_i indicates the number of nodes with i interfaces) [2]. In Figure 6, there are 2 nodes with 3 interfaces ($ID_3 = 2$), 2 nodes with 2 interfaces ($ID_2 = 2$), and 2 nodes with 1 interface ($ID_1 = 2$). Subsequently, we can compute the scaling coefficient $\mathbf{A_I}$ of the interface distribution.

Once the interface distribution is determined based on Algorithm 1, the number of interfaces (i.e., I) can be calculated from $\mathbf{I} = \sum_{\mathbf{i}} \mathbf{i} \cdot \mathbf{ID_i}$. The number of interfaces on all nodes I is equal to the sum of subnetwork sizes (i.e., S) so that all nodes and subnetworks are interconnected. That is

$$\sum_i i \cdot ID_i = \sum_j j \cdot SD_j \tag{1}$$

Subsequently, for a given subnetwork distribution exponent $\alpha_{\mathbf{S}}$, we can determine the scaling coefficient $\mathbf{A_D}$ of the subnetwork distribution.

4.3 Dependence of Distributions

In this section, we analyze the dependency of degree distribution to the interface and subnetwork distributions when all are power-laws. As the degree distribution is determined from the 1-mode projection of the nodes in the 2-mode graph, it is dependent on the underlying distributions of the 2-mode graph.

Once the power-law exponents of interface distribution (i.e., $\alpha_{\mathbf{I}}$) and subnetwork distribution (i.e., $\alpha_{\mathbf{S}}$) are determined, we can compute the average degree (i.e., $< k >$) in the network as follows. A subnetwork of size j, by definition, connects j nodes in the one-hop distance. Hence, once a node connects to this subnetwork, its degree k increases by $\mathbf{j-1}$. Subsequently, the total degree contribution of the subnetwork to the network is $\mathbf{j} \cdot (\mathbf{j-1})$. When we consider all of the subnetworks in the network, the total degree $\sum k$ can be calculated as $\sum_{\mathbf{j}} \mathbf{SD_j} \cdot \mathbf{j} \cdot (\mathbf{j-1})$ where SD_j indicates the number of subnetworks with j devices. Dividing this value by the number of nodes (i.e., N) gives the average degree $< k >$ of the network as follows.

$$< k > \ = \{ \sum_j SD_j \cdot j \cdot (j-1) \ \}/N \tag{2}$$

Similarly, the degree distribution can be utilized to calculate the number of nodes. That is, $\sum_{\mathbf{k}} \mathbf{k} \cdot \mathbf{DD_k} = < k > \cdot N$ where DD_k indicates the number of nodes with degree k. Hence,

$$< k > \ = \{ \sum_k k \cdot DD_k \ \}/N \tag{3}$$

Additionally, for a given power law exponent α and a number of nodes N, we can calculate the A coefficient. Therefore, the power law exponent of the degree distribution (i.e., α_D) can be calculated for a given set of N, α_I and α_S parameters using the equations 2-3 and the power-law formula of $\mathbf{F_i} = \mathbf{A}\mathbf{i}^{-\alpha}$.

Figure 7 presents the power-law exponent of the degree distribution α_D with respect to a given pair of subnetwork α_S and interface α_I distributions assuming ideal power-law distributions. Note that subnetwok distribution assumes that there is no subnetwork of size 1 (i.e., $SD_1 = 0$) and hence the plot is not symmetrical. We calculate the power-law exponent of the degree distribution α_D for all interface $\alpha_{\mathbf{I}}$ and subnetwork $\alpha_{\mathbf{S}}$ combinations with an increment of 0.1. Note that some of the combinations in the vicinity of 1.0 x 1.0 have a $\alpha_{\mathbf{D}} = 0$ (shown as black color) since they are infeasible. In a power-law distribution, when $1 < \alpha < 2$, the first moment (i.e., the average $< k >$) is infinite along with all the higher moments [40]. Similarly, when $2 < \alpha < 3$, the first moment is finite, but the second (i.e., the variance) and higher

Fig 7. Correlation among the distributions

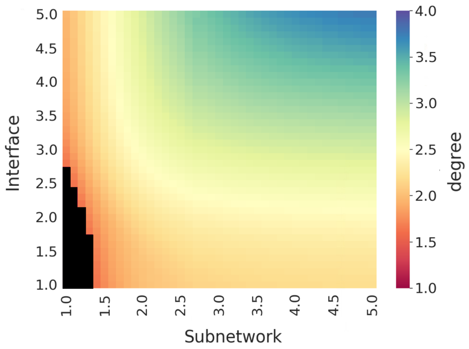

moments are infinite. Since such distributions contain extremely large values [41], obtaining a feasible configuration of interface and subnetworks (see Figure 6) becomes impossible.

4.4 Network Connectivity

Utilizing pure probabilistic generation methods after assigning certain target degree to nodes performs poorly in terms of connectivity [32]. During our experiments, we observed that random matching of interfaces to subnetworks result in disconnected graphs and that the giant component shrinks as the subnetwork and interface distributions become steeper. In this section, we analyze issues in ensuring connectivity between all subnetworks and nodes during the 2-mode graph generation.

Generating a connected network with power-law degree distribution is not possible for all $\alpha_{\mathbf{I}}$ and $\alpha_{\mathbf{S}}$ pairs. Intuitively, if the ratio of single interface routers increases, it becomes harder or even impossible to generate a connected network. Similarly, having subnetworks with only two interfaces limit the number of configurations. Overall, every node with more than one interface can utilize its first interface to attach to the current giant component and each of the other interfaces to attach a new subnetwork. Hence, the condition that guarantees the existence of a connected configuration can be formulated as

$$S \leq 1 + \sum_{i=2} (i-1) \cdot ID_i \tag{4}$$

where S indicates the total number of interfaces in all of the generated subnetworks.

Figure 8 plots the connectivity with respect to the power-law exponents of the subnetwork and interface distribution where the black line indicates above which connected networks cannot be generated. Similarly, the red line shows the region below which networks are infeasible irrespective of connectivity as discussed in Section 4.3. The figure also presents the power-law exponents of the interface and subnetwork distributions of the AS sampled by ASM [22] (also employed in the evaluations of Section 5.2) and observe them to be within the feasible region. When the interface and subnetwork distribution exponents are above the black line, there is no connected graph satisfying both power-law distributions.

As the slopes become steeper, the ratio of single interface nodes and/or ratio of smaller subnetworks increases, and eventually connectivity becomes infeasible. Analysis of the contour and the axes reveal that connectivity is affected more by the subnetwork distribution compared to the interface distribution. Note that, as the networks are generated through a random process and the obtained power-laws are not perfect the boundaries are not hard boundaries and one may obtain a connected network with

Fig 8. Feasible α_I and α_S Exponents for a Connected Network

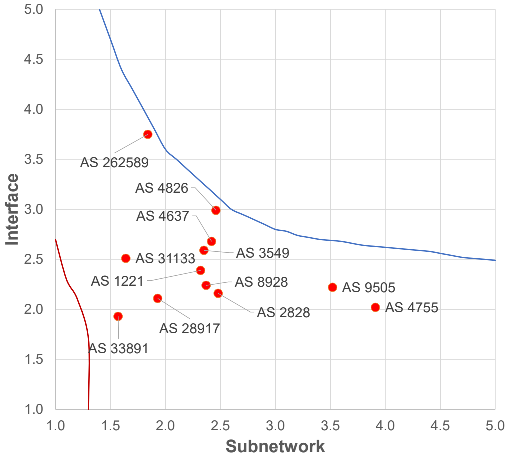

power-laws above the line. However, it becomes harder to find a connected network configuration as power-law exponents are closer to either boundary.

4.5 Generation Methodology

Precise matching of the distributions can be challenging to achieve due to the discrete nature of the graphs [40]. After calculating all distributions using Algorithm 1, we generate vertices of the bipartite graph without any edges. We assign the interface count of each *node* and the size of each *subnetwork* according to the previously calculated $\mathbf{ID_i}$ and $\mathbf{SD_j}$, respectively. We mix the order of the nodes and subnetworks to eliminate bias (i.e., obtain non-assortative graphs). If both subnetworks and nodes are ordered with respect to their size, the assortative mixing of the resulting graph would be high [42]. However, our analysis of measured AS networks indicate non-assortative connectivity in terms of attachment between low/high interfaces and subnetworks.

Algorithm 2 provides the pseudo-code for the network generation from a given set of Nodes and Subnetworks that are produced with the Algorithm 1 using the interface and subnetwork distributions, respectively. In order to ensure connectivity (see Section 4.4), we employ a *Connected* set of subnetworks that are connected to the main component so far. As the network is modeled as a bipartite graph, SubNetG grows the network expanding the connected component to have a path between any subnetwork pairs. The design relies on the idea that at least one interface of each node is connected to a subnetwork that is already part of the connected component, while the rest of the interfaces of the node are connected to random subnetworks.

After initializing *Edges* to an empty set (Line 1), we assign a random Subnetwork to the *Connected* component (Line 2). The outer loop in Line 3 iterates over all *Nodes* that were randomly sorted and the inner loop (Line 10) iterates over each interface of the node (i.e., $Nodes_i$) after the first one (i.e., $Nodes_i^1$) is connected to the *Connected* component (Lines 4-8). A random subnetwork is selected to be connected (Lines 11-16) and the selected subnetwork is appended to the *Connected* component (Line 17). Lines 5-8 and Lines 12-16 ensure that the selected subnetwork has room for a new node connection, and there is not already an edge between the node and subnetwork. If the subnetwork is full, it is removed from the respective list so that it is not redundantly selected (lines 6-7 and 14-15). Finally, the selected subnetwork is connected to the node's interface (Line 18). The algorithm terminates when all interfaces of all nodes have been considered for attaching to the subnetworks. Note that in practice we also

Algorithm 2: GenerateNetwork(*Nodes, Subnetworks*)

```
1  Edges[ ] ← { }
2  Connected[ ] ← { random(Subnetworks) }
3  for i ← 1 ... |Nodes| do
4      subnetwork ← random(Connected)
5      while Full(subnetwork) do
6          Connected ← Connected − {subnetwork}
7          Subnetworks ← Subnetworks − {subnetwork}
8          subnetwork ← random(Connected)
9      Edges ← Edges ∪ {edge(Nodes_i^1, subnetwork)}
10     for j ← 2 ... Nodes_i^IC do
11         subnetwork ← random(Subnetworks)
12         while Full(subnetwork) or ∃ edge(Nodes_i, subnetwork) ∈ Edges do
13             if Full(subnetwork) then
14                 Subnetworks ← Subnetworks − {subnetwork}
15                 Connected ← Connected − {subnetwork}
16             subnetwork ← random(Subnetworks)
17         Connected ← Connected ∪ {subnetwork}
18         Edges ← Edges ∪ {edge(Nodes_i^j, subnetwork)}
```

need to check for the parameters to ensure feasibility before execution and consider the lack of subnetworks especially in the *Connected* component during network configuration.

Figure 9 presents the execution of the Algorithm 2 on the sample bipartite graph in Figure 6. Note that subnetworks and routers are randomly sorted to remove assortative linking. Figure 9-**a** shows the state of the graph at the end of line 2 assuming S_2 is randomly selected as the initially *Connected* subnetwork. Line 3 selects the first router R_1 in the list and line 4 selects S_2 as it is the only subnetwork in the *Connected* component. AS S_2 has room for attachment, lines 5-8 are skipped. Then, line 9 adds an edge between the first interface of R_1^1 and S_2 as shown in Figure 9-**b**. Subsequently, line 10 picks the second interface of R_1, and line 11 picks a random subnetwork, assume S_4. As there are non-connected interfaces of S_2, lines 12-16 are skipped. Note that, the

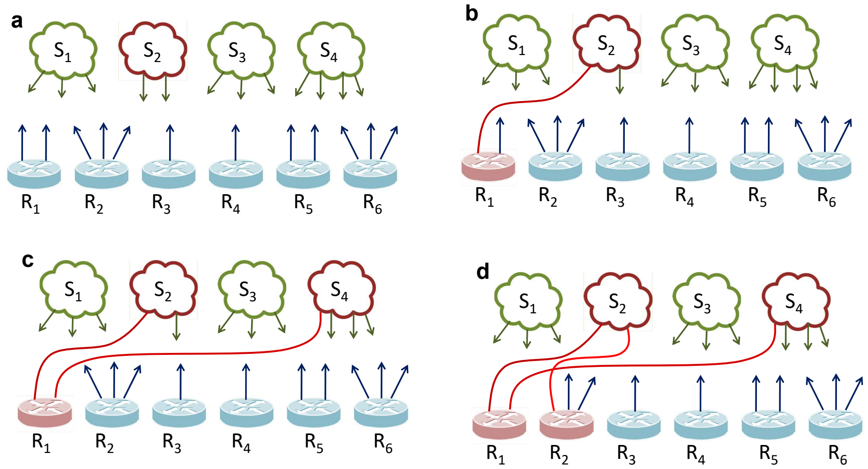

Fig 9. Iteration of Algorithm 2 on a Sample Network

algorithm could have selected a subnetwork already in the *Connected*. Line 17 adds the subnetworks to the *Connected* and line 18 adds and edge between the second interface R_1^2 and S_4 as shown in Figure 9-**c**. Finally, algorithm loops back to second router R_2 in line 3, and randomly selects subnetwork S_2 in line 4 to be connected as shown in Figure 9-**d**. Note that as S_2 is now full, it will be removed from the *Connected* and *Subnetworks* when it is selected in line 4.

The time complexity of the algorithm is $O(I+S)$ where I is the total number of interfaces in the network, and S is the number of subnetworks. While there are two outer loops at line 3-4, each node interface is processed only once. Likewise, the internal loops at lines 7 and 12 are executed only once for each subnetwork to be removed from the list when it becomes full.

5 Evaluation

In this section, we present samples of 2-mode power-law network generation with SubNetG. In Section 5.1, we analyze whether current topology generators reflect link-level subnetwork characteristic. In Section 5.2, we evaluate the data we obtained from large backbone Autonomous Systems sampled across the Internet. In Section 5.3, we show the results for the synthetic topologies that we generate using SubNetG.

5.1 Analysis of Network Generators

In this section, we assess whether network generators capture the link-level characteristic (i.e., subnetwork connectivity) of the Internet topology. As commonly employed network generators produce 1-mode graphs [43], we transform the 2-mode network of link-level Internet (i.e., subnetworks and nodes) into a 1-mode network between routers. Hence, we model subnetworks as cliques between all attached nodes, as shown in Figure 10. Note that this estimation is not perfect as neighboring subnetworks (such as three point-to-point links between the same triple of nodes) may incorrectly be assumed as a single subnetwork (i.e., a three node subnetwork).

In order to analyze the clique distribution, we sampled the subnetwork based Internet2 backbone topology [37] . Note that, even though router-level topology of other networks are publicly shared, they do not provide the subnetwork information that is needed for a ground truth comparison. We convert the subnetwork topology to a point-to-point graph by replacing multi-access links of each subnetwork with a clique of links among the subnetwork nodes. Finally, we run a clique search on the graph and compare the distributions with the actual *Internet2 Subnetwork distribution* to analyze how successful the clique search approach captures the multi-access links.

Internet2 Subnetwork curve in Figure 11 illustrates the subnetwork distribution of the Internet2 backbone. The result of clique search is shown as *the Internet2 Clique* curve. We observe a slight difference between the *subnetwork* and *clique* distributions, i.e., at 2 and 3. In clique search, all point-to-point triangles are assumed to be a multi-access link with three nodes. Note that, similar incorrect assumptions in larger cliques have a negligible probability of occurrence as this will require all nodes in both subnetworks to be attached to each other. We also utilize the *Orbis topology*

Fig 10. Multi-access vs. point-to-point links

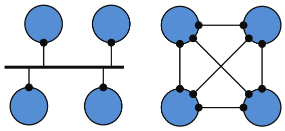

Fig 11. Clique size distributions of Internet2

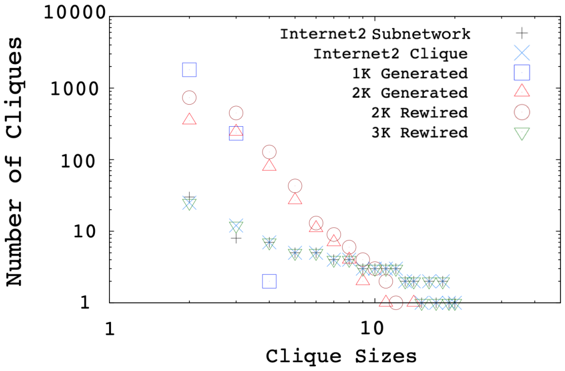

generator [33], to produce the same size graph with *Internet2 Clique* as the reference graph. Orbis can produce synthetic topologies of any size with the exact 1k or 2k distribution (marked as *Generated* in Figure 11), and rewire the original graph while preserving the 2k or 3k distributions (marked as *Rewired* in the Figure). Although the reference Internet2 graph includes up to 20-cliques, 1k or 2k synthetic topologies do not preserve the clique distribution. We observe that 3k rewiring preserves the clique distribution, but a close examination revealed that cliques larger than 3 were not rewired and remained intact.

Finally, we produced topologies replicating the characteristics of sample AS networks in Table 2. We generated synthetic graphs using Inet [30] and BRITE [6] with both of the Waxman and Barabasi-Albert (BA) models. For BA preferential attachment model, BRITE uses an m parameter to indicate the number of connections a new node makes. As the m value is increased, network density increases. However, this did not result in a significant increase in the size of cliques in the topology. Figure 12 shows the clique distributions of a sample AS 8928. As shown in the figure, both the number of cliques and the clique sizes in the real topology is significantly larger than the ones in the generated topologies. The generated topologies have clique sizes up to 20, but as seen from the figure, measured topologies of the similar sizes can have clique sizes up to 200. Inet produces largest cliques among other generators but it has a completely different distribution compared to the measurements.

Both *the Internet2 Subnetwork* curve in Figure 11 and clique sizes and clique distribution of the measured AS in Figure 12 illustrate the frequencies of multi-access links in the Internet2 and real Internet topology, hence it shows the existence of subnetworks in the backbone. Overall, we observe none of the analyzed generators capture the subnetwork characteristics of the sampled backbone Internet topologies.

Fig 12. Clique Size Distribution of the AS 8928

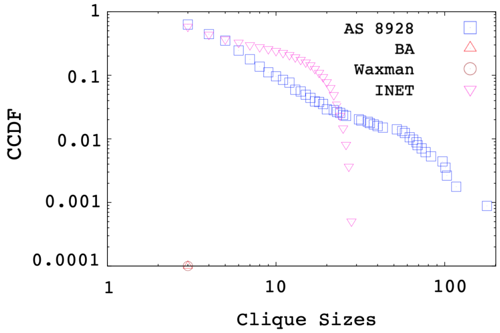

5.2 Sample Network Topologies

In this section, we analyze the topological characteristics of sample AS networks obtained from measurements of the Internet backbone via Autonomous System Mapper (ASM) [22]. ASM collects partial traces to every observed IP address of an AS from all border routers it could identify for the AS. After filtering trace anomalies such as loops and bounce-backs [44], ASM identifies subnetworks [45], IP aliases [46], and unresponsive routers [47] to infer the underlying link-level network of the AS. Even though ASM collects the most comprehensive snapshot of backbone AS, there may still be unmapped regions of the network. While public measurement platforms such as CAIDA Ark [48] and RIPE Atlas [49] provide samples of the Internet backbone, they do not comprehensively map all interface IPs of an AS. In a previous study [43], we had relied on such public measurement platforms [22, 48, 50] to estimate the power-law parameters. In this study, we realized that their samples yield power-law parameters that were not within the feasible range as discussed in Section 4.4. Hence, we deployed ASM to better capture link-level connectivity of AS topologies.

Interface distribution presents the histogram of systems (e.g., router and server) with a given number of interfaces. The number of interfaces on a system is typically equal to the number of IP aliases for that system. ASM utilizes MIDAR [51] and ASIAR [52] tools to resolve IP aliases in the sample topologies. Figure 13 present the interface distributions of three sample AS. The power-law exponent value of most of the measured AS vary between 2 and 3, as shown in Table 2. We defaulted the interface distribution of SubNetG to the average of sampled AS, i.e., $\alpha = \mathbf{2.5}$.

Subnetwork distribution presents a histogram of the subnetworks with a specific size, i.e., the number of interfaces attached to the subnetwork [38]. For a given $\mathbf{i}$, $\mathbf{SD_i}$ indicates the number of subnetworks with $\mathbf{i}$ attached systems. Note that the minimum $\mathbf{i}$ is 2, as there should be at least two interfaces attached to a subnetwork. This metric complements the interface distribution, where the total number of interfaces is equal to the sum of the subnetwork sizes. We use ASIAR [52] to infer the subnetworks of the AS based on the BGP announcements of the AS. Figure 14 presents the subnetwork distributions of three sample AS. The power-law exponent value of the sampled ASes is

Fig 13. Interface distribution of AS 1221, AS 8928 and AS 2828

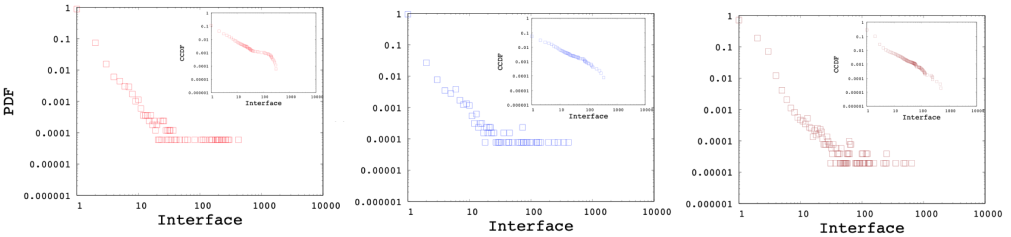

Fig 14. Subnetwork distribution of AS 1221, AS 8928 and AS 2828

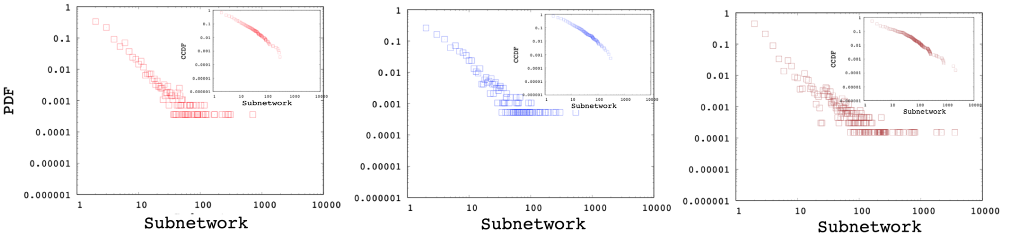

Fig 15. Degree distribution of AS 1221, AS 8928 and AS 2828

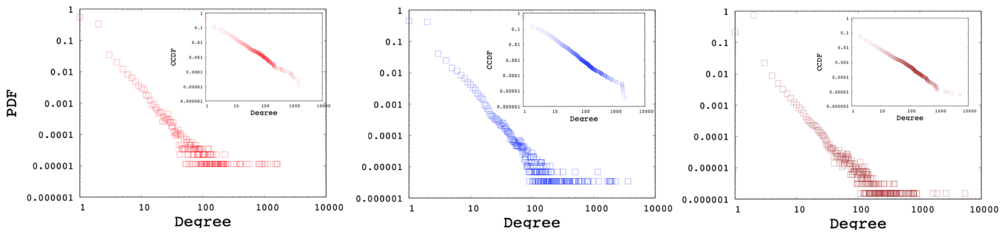

Table 2. Power-law exponents for sample backbone AS

**AS**	**1221**	**2828**	**3549**	**4637**	**4755**	**4826**	**8928**	**9505**	**28917**	**31133**	**33891**	**262589**	mean
α_I measured	2.39	2.16	2.59	2.68	2.02	2.99	2.24	2.22	2.11	2.51	1.93	3.75	2.47
α_S measured	2.32	2.48	2.35	2.42	3.91	2.46	2.37	3.52	1.93	1.64	1.57	1.84	2.40
α_D measured	2.52	2.42	2.31	2.74	2.16	2.62	2.59	3.23	2.19	2.29	2.27	2.12	2.45
α_D expected	2.45	2.41	2.54	2.58	2.44	2.71	2.39	2.50	2.20	2.26	1.95	2.56	2.42
α_D generated	2.35	2.31	2.68	2.64	2.07	3.22	2.29	2.49	2.17	2.76	2.04	3.30	2.53

between 1.5 and 4, as shown in Table 2. We consider the average of samples, i.e., $\alpha = \mathbf{2.4}$, as the default while generating synthetic topologies with SubNetG.

Degree distribution is the most utilized metric to characterize a network. Degree distribution represents a histogram of the nodes with a certain degree and gives insight into the structure of the network. For each node, its degree is computed from the number of nodes that are within one-hop distance. For the sampled backbone AS, the power-law exponent α value is mostly within 2 and 3 range and has an average of 2.45. Figure 15 shows the degree distributions of three sample AS networks.

Table 2 presents the power-law exponent for the Interface, Subnet, and Degree distributions of 12 AS mapped by ASM. As research has shown that visual verification of the linearity in the logarithmic scale may be misleading, we utilize [41] for fitting and verifying the observed power-laws. Note that, as the empirical data is not ideal power-law distributions, the α_D *measured* and α_D *expected* differ for individual AS measurements (such as in AS 9505, 33891 and 262589). Overall, the median and mean difference between the expected and measured α_D is 0.07 and 0.09, respectively. Similarly, generated networks employ a random configuration of subnetworks and node interfaces after establishing a minimum spanning tree to ensure connectivity, and hence α_D *generated* may differ from both as seen with AS 4826, 31133 and 262589. Overall, the median and mean difference between the expected and generated α_D is 0.04 and 0.08, respectively, indicating that generated networks are closer to the expected distributions with the exception of a few outliers. One may generate new networks until a configuration within a threshold of α_D is obtained. Generated networks are further analyzed in the next Section.

5.3 Synthetic Topologies

In this section, we analyze synthetic topologies generated with the interface and subnetwork distributions of the measured backbone AS. While SubNetG matches the measured power-law exponents of the interface α_I and subnetwork α_S distributions, it does not directly match the degree distribution α_D. We observe that the resulting power-law exponents of the degree distributions to be similar to the genuine networks as presented in the Table 2. Furthermore Figure 16, 17, and 18 show the comparison of measured and generated distributions for the Subnet, Interface and Degree of three

sample AS 1221, 2828, and 8929, respectively. Red dots show the values from the genuine AS measurements while black dots show the values of the synthetic network generated by SubNetG. We observe that, the generated interface and subnetwork distributions are different from the measured distributions even though the power-law exponents are the same. Particularly, the CCDF of measured distributions seem to contain greater perturbations, which is expected in empirical data [41].

Additionally, we generate synthetic topologies with 1K, 10K, 100K, and 1M nodes. Based on the averages of AS measurements presented in Section 3, we set $\alpha_{\mathbf{ID}} = \mathbf{2.5}$ and $\alpha_{\mathbf{SD}} = \mathbf{2.4}$. Figures 19 present the interface, subnetwork, and degree distributions, of the generated networks. Even though the generation method did not consider the degree distribution directly, the resulting degree distributions are power-law distributions with an average exponent of $\alpha_{\mathbf{DD}} = \mathbf{2.77, 2.75, 2.58,}$ and $\mathbf{2.53}$, respectively for 1K, 10K, 100K, and 1M nodes.

Although sample topologies presented in this study use data obtained from ASM [22], the presented algorithm is independent of the specific data set and can produce synthetic topologies with any feasible set of distribution. Generation parameters $\alpha_{\mathbf{DD}}$, $\alpha_{\mathbf{ID}}$ and $\alpha_{\mathbf{SD}}$ can be selected from any measurement dataset and ported as the reference topology. Moreover, the user can supply any feasible (for feasible parameter space) set of parameters to be matched. Overall, we observe that produced networks have a similar interface, subnetwork, and degree distributions to the genuine topology they are modeled after.

Fig 16. Distributions of a synthetic network based on AS 1221

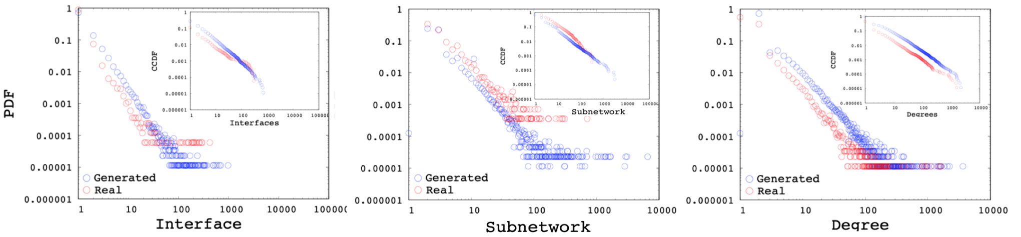

Fig 17. Distributions of a synthetic network based on AS 2828

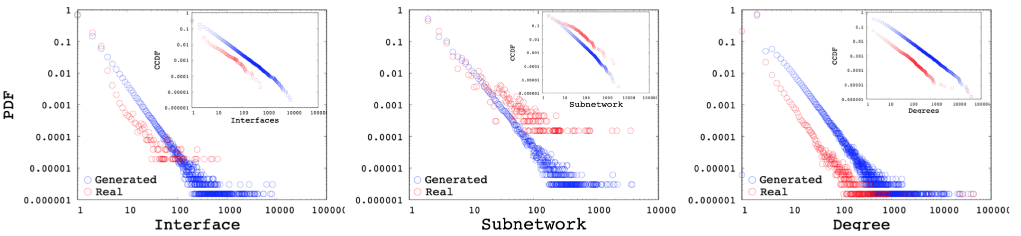

Fig 18. Distributions of a synthetic network based on AS 8929

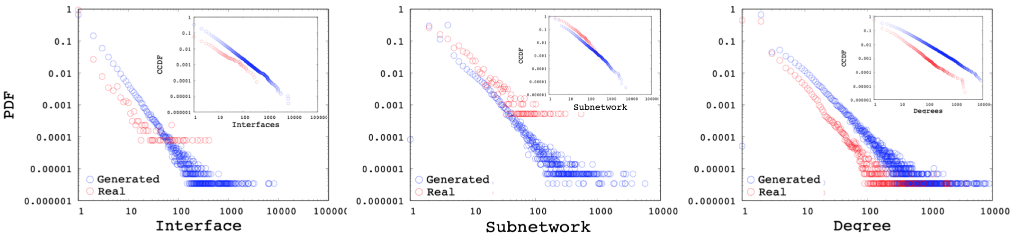

Fig 19. Interface, Subnetwork and Degree distribution of generated networks with 1K, 10K, 100K and 1M nodes

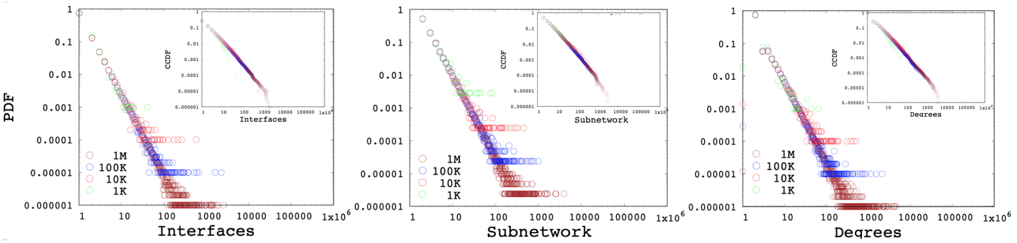

6 Conclusion

Currently, synthetic network generators for the Internet topology ignore the multi-access links and model the network as consisting of point-to-point links. However, multi-access links such as FDDI ring, and Ethernet are widely deployed as link-layer technologies at the backbone networks. To assess the need for 2-mode modeling, we analyzed the impact of subnetworks on the Internet topologies by comparative graph structure analysis of current network topology generators and performed comparative network simulations. Our analyses on the previous network topology generators revealed that neither the subgraph structures nor the bandwidth related characteristics of the Internet topology are represented by the generated graphs. Additionally, we analyzed the interface and subnetwork size distributions of sample backbone AS in addition to the degree distribution that the current power-law based topology generators focus on. In our analysis of top ranked backbone AS, we observe that both subnetwork and interface distributions occasionally exhibit power-law characteristics similar to the degree distribution.

We introduced SubNetwork Generator (SubNetG) that captures both the link-level interface and subnetwork distributions and the network-level degree distribution. We showed that the degree distribution is uniquely defined for a given pair of subnetwork and interface distributions that are ideal power-laws. We also showed the necessary condition for obtaining a connected graph with all distributions being a power-law. Note that, the generation parameters (i.e., interface and subnetwork distributions and network size) can be estimated based on the measurement results or provided by the user. Finally, we present synthetic networks and show that the SubNetG captures subnetwork characteristics of the link-layer Internet topologies as well the as degree distribution. The SubNetG can be used for generating synthetic network topologies at link-level, which can be utilized for simulating network protocols for more realistic link-layer behavior (i.e., multi-access links) as well as analysis of link-layer topologies that reflect the interaction between layer 2 (i.e., subnetworks) and layer 3 (i.e., routers). As future work, the SubNetG algorithm can be improved to eliminate potential outliers, consider router-level metrics such as rich-clubs that would represent network cores, and generate subnetworks with other distributions such as exponential, log-normal, and Weibull.

7 Acknowledgments

We would like to thank Mehmet Burak Akgun for the initial effort on this work.

References

1. R. Pastor-Satorras, A. Vespignani, Evolution and Structure of the Internet: A Statistical Physics Approach, Cambridge, Cambridge University Press.,2004, doi:10.1017/CBO9780511610905

2. M. Newman, Networks: An Introduction, Oxford University Press, Inc., 2010

3. M. H. Gunes, S. Bilir, K. Sarac, T. Korkmaz, A measurement study on overhead distribution of value-added internet services, Computer Networks, Volume 51, Issue 14, 2007, Pages 4153-4173, doi:10.1016/j.comnet.2007.05.001

4. L. Cheng, N.C. Hutchinson, M. R. Ito, RealNet: A Topology Generator Based on Real Internet Topology, 22nd International Conference on Advanced Information Networking and Applications - Workshops (AINA workshops 2008), Okinawa, 2008, pp. 526-532, doi: 10.1109/WAINA.2008.66

5. N. Hidaka, S. Arakawa, M. Murata, A Modeling Method for ISP Topologies Based on Network-Cost Optimization, Fourth International Conference on Autonomic and Autonomous Systems (ICAS'08), Gosier, 2008, pp. 169-174, doi: 10.1109/ICAS.2008.7

6. A. Medina, A. Lakhina, I. Matta, J. Byers, BRITE: Universal Topology Generation from a User's Perspective, 2001, View on NCSTRL, Boston University, Boston, MA, USA

7. C. Labovitz, A. Ahuja, R. Wattenhofer, S. Venkatachary, The impact of Internet policy and topology on delayed routing convergence, Proceedings IEEE INFOCOM 2001. Conference on Computer Communications. Twentieth Annual Joint Conference of the IEEE Computer and Communications Society, Anchorage, AK, USA, 2001, pp. 537-546 vol.1, doi: 10.1109/INFCOM.2001.916775

8. M. H. Gunes, K. Sarac, Importance of IP Alias Resolution in Sampling Internet Topologies, 2007 IEEE Global Internet Symposium, Anchorage, AK, 2007, pp. 19-24, doi: 10.1109/GI.2007.4301425

9. Z. Wang, A. Scaglione, R. J. Thomas, On Modeling Random Topology Power Grids for Testing Decentralized Network Control Strategies, IFAC Proceedings Volumes, Volume 42, Issue 20,2009, Pages 114-119, doi:10.3182/20090924-3-IT-4005.00020

10. C. Giudicianni, A. Di Nardo, M. Di Natale, R. Greco, G. F. Santonastaso, A. Scala, 2018, Topological Taxonomy of Water Distribution Networks, Water 10, no. 4, 444

11. R. Albert, H. Jeong, A. Barabási, Error and attack tolerance of complex networks. Nature 406, 378–382 (2000), doi:10.1038/35019019

12. J. Kleinberg, Navigation in a small world, Nature 406, 845 (2000), doi:10.1038/35022643

13. P. Erdos, A. Renyi, On random graphs. I, Publ. Math. Debrecen, 1959, vol. 6, pages,290–297

14. D. J. Watts , S. H. Strogatz, Collective dynamics of 'small-world' networks, Nature, 1998 Jun 4,393(6684):440-2, doi: 10.1038/30918

15. M. Latapy, C. Magnien, Complex Network Measurements: Estimating the Relevance of Observed Properties, IEEE INFOCOM 2008 - The 27th Conference on Computer Communications, Phoenix, AZ, 2008, pp. 1660-1668, doi: 10.1109/INFOCOM.2008.227

16. S. A. Hale, Global connectivity and multilinguals in the Twitter network, In Proceedings of the SIGCHI Conference on Human Factors in Computing Systems (CHI '14), 2014, Association for Computing Machinery, New York, NY, USA, 833–842. doi:10.1145/2556288.2557203

17. K. Komurov, M. H. Gunes, M. A. White, Fine-scale dissection of functional protein network organization by statistical network analysis, PloS one 4.6 (2009): e6017

18. H. Kardes, M. Gunes, T. Oz, Cheleby: A subnet-level internet topology mapping system, 2012 Fourth International Conference on Communication Systems and Networks (COMSNETS 2012), Bangalore, 2012, pp. 1-10, doi: 10.1109/COMSNETS.2012.6151326

19. M. E. Tozal, K. Sarac, TraceNET: an internet topology data collector, In Proceedings of the 10th ACM SIGCOMM conference on Internet measurement (IMC '10), 2010, Association for Computing Machinery, New York, NY, USA, 356–368. doi:10.1145/1879141.1879188

20. M. A. Canbaz, K. Bakhshaliyev, M. H. Gunes, Router-level topologies of autonomous systems, International Workshop onComplex Networks, 2018, doi:10.1007/978-3-319-73198-8_21

21. M. Faloutsos, P. Faloutsos, C. Faloutsos, On power-law relationships of the Internet topology, SIGCOMM Comput. Commun. Rev., ACM , vol. 29,pages 251–262, August ,1999,New York, NY, USA

22. Autonomous System Mapper tool, http://im.cse.unr.edu/, (accessed: 8.20.2020)

23. M. B. Doar, A better model for generating test networks, Proceedings of GLOBECOM'96. 1996 IEEE Global Telecommunications Conference, London, UK, 1996, pp. 86-93, doi: 10.1109/GLOCOM.1996.586131

24. M. Thomas, E. W. Zegura, Generation and Analysis of Random Graphs to Model Internetworks, College of Computing, 1994

25. B. Quoitin, Towards more representative internet topologies, Universit Catholique de Louvain; Tech. Rep. 2010

26. R. Govindan, H. Tangmunarunkit, Heuristics for Internet map discovery, Proceedings IEEE INFOCOM 2000. Conference on Computer Communications. Nineteenth Annual Joint Conference of the IEEE Computer and Communications Societies, Tel Aviv, Israel, 2000, pp. 1371-1380 vol.3, doi: 10.1109/INFCOM.2000.832534

27. A. Medina, I. Matta, J. Byers, On the origin of power laws in Internet topologies, SIGCOMM Comput. Commun. Rev. 30, 2000, 18–28. doi:10.1145/505680.505683

28. P. L. Krapivsky, S. Redner, F. Leyvraz, Connectivity of Growing Random Networks, Physical Review Letters,Nov, 2000, American Physical Society, vol 85, pages 4629-4632; doi:10.1103/physrevlett.85.4629

29. A. L. Barabasi, R. Albert, Emergence of scaling in random networks, Science, 1999, vol 286, page 509, American Association for the Advancement of Science, doi:10.1126/science.286.5439.509

30. J. Winick, S. Jamin, Inet-3.0: Internet topology generator, Technical Report CSE-TR-456-02, University of Michigan, 2002

31. T. Lappas, K. Pelechrinis, M. Faloutsos and S. V. Krishnamurthy, A simple conceptual generator for the Internet graph, 2010 17th IEEE Workshop on Local Metropolitan Area Networks (LANMAN), Long Branch, NJ, 2010, pp. 1-6, doi: 10.1109/LANMAN.2010.5507153

32. P. Mahadevan, D. Krioukov, K. Fall, A. Vahdat,Systematic topology analysis and generation using degree correlations, SIGCOMM Comput. Commun. Rev. 36, 2006, 135–146. doi:10.1145/1151659.1159930

33. P. Mahadevan, C. Hubble, D. Krioukov, B. Huffaker, A. Vahdat, Orbis: rescaling degree correlations to generate annotated internet topologies. SIGCOMM Comput. Commun. Rev. 37, 2007, 325–336. doi:10.1145/1282427.1282417

34. Internet topology at router- and AS-levels, and the dual router+AS Internet topology generator, https://www.caida.org/research/topology/generator, (accessed: 4.18.2020)

35. X. Dimitropoulos, D. Krioukov, A. Vahdat, G. Riley, Graph Annotations in Modeling Complex Network Topologies, ACM Transactions on Modeling and Computer Simulation (TOMACS), Oct, 2009, vol 19, page 17

36. D. Krioukov, F. Papadopoulos, M. Kitsak, A. Vahdat, M. Boguñá, Hyperbolic Geometry of Complex Networks, Physical Review E, Oct,2010, vol 82

37. Internet2 community, https://www.internet2.edu/, (accessed: 07.25.2020)

38. M. Gunes, K. Sarac, Inferring Subnets in Router-level Topology Collection Studies, ACM SIGCOMM Internet Measurement Conference (IMC),San Diego, CA, October, 2007

39. Network Simulator - ns3, https://www.nsnam.org/, (accessed: 07.25.2020)

40. M. E. J. Newman, Power laws, Pareto distributions and Zipf's law, Contemporary physics, Taylor & Francis, 2005, vol. 46, pages 323–351

41. A. Clauset, C. R. Shalizi, M. E. J. Newman, Power-Law Distributions in Empirical Data, SIAM Rev.,Society for Industrial and Applied Mathematics,November 2009,vol. 51, pages 661–703, Philadelphia, PA, USA

42. M. E. J. Newman, Assortative Mixing in Networks, Physical Review Letters, American Physical Society, Oct, 2002, doi:10.1103/physrevlett.89.208701

43. M. B. Akgun, M. H. Gunes, Impact of Multi-access Links on the Internet Topology Modeling, 2012 IEEE 20th International Symposium on Modeling, Analysis and Simulation of Computer and Telecommunication Systems, IEEE Computer Society,2013, vol. 0, pages 399-403, Los Alamitos, CA, USA

44. K. Bakhshaliyev, M. A. Canbaz, M. H. Gunes, Investigating Characteristics of Internet Paths, ACM Transactions on Modeling and Performance Evaluation of Computing System (TOMPECS), ACM, 2019

45. H. Kardes, T. Oz, M. H. Gunes, Cheleby: A Subnet-level Internet Topology Mapping System, COMSNETS, January, 2011

46. M.H. Gunes, K. Sarac, Resolving IP Aliases in Building Traceroute-Based Internet Maps, Networking, IEEE/ACM Transactions, December 2009, vol. 17, pages 1738 -1751

47. H. Kardes, M. H. Gunes, K. Sarac, Graph Based Induction of unresponsive routers in Internet topologies, Computer Networks, Elsevier, 2015, vol. 81, pages 178–200

48. M. Luckie, Y. Hyun, B. Huffaker, Traceroute probe method and forward IP path inference, IMC '08, Proceedings of the 8th ACM SIGCOMM conference on Internet measurement, ACM, 2008, Vouliagmeni, Greece, pages 311–324

49. RIPE, NCC, RIPE Atlas. https://atlas.ripe.net/, (accessed: 8.20.2020)

50. iPlane,http://iplane.cs.washington.edu/, (accessed: 8.20.2020)

51. midar tool, http://www.caida.org/tools/measurement/midar, (accessed: 8.20.2020)

52. A. Aksoy, M. H. Gunes, ASIAR: Analytical Subnetwork and IP Alias Resolution for Network Tomography using Path Traces, Elsevier Computer Networks, 2019, (under revision)
