# Supplementary figures and images for: Generation of 2-mode scale-free graphs for link-level internet topology modeling
Source: PLoS One. 2020 Nov 9;15(11):e0240100. doi: 10.1371/journal.pone.0240100 (PMC7652253; doi:10.1371/journal.pone.0240100)

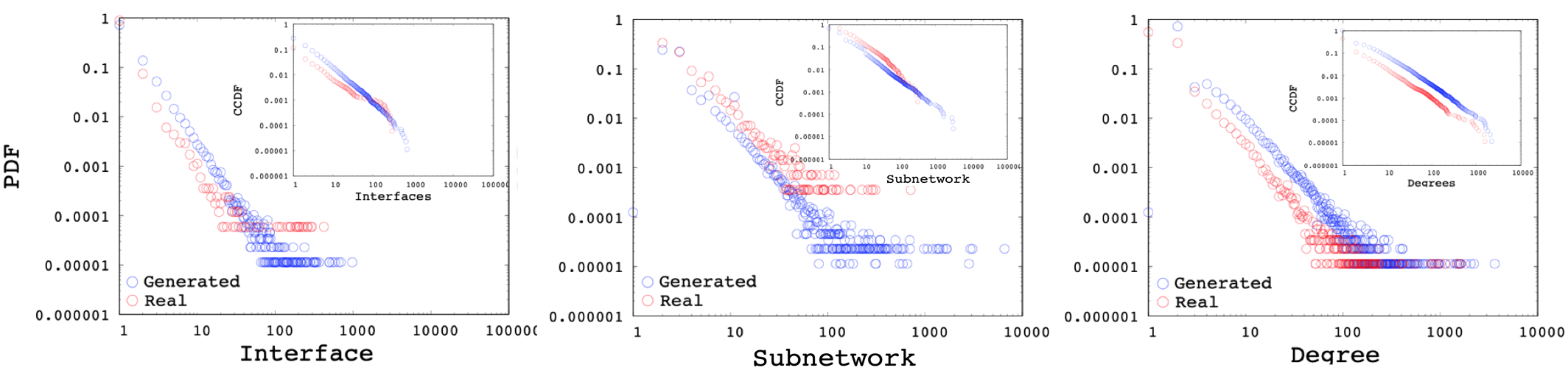

Supplement: S1 File — (ZIP) [file pone.0240100.s001.zip › SubNetG_ revision/figures/1221.png]

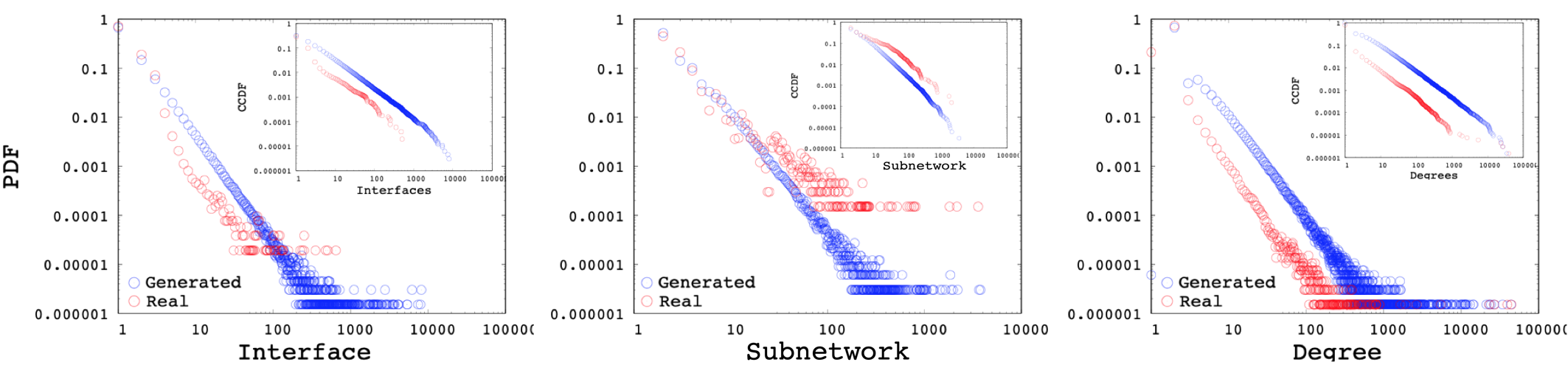

Supplement: S1 File — (ZIP) [file pone.0240100.s001.zip › SubNetG_ revision/figures/2828.png]

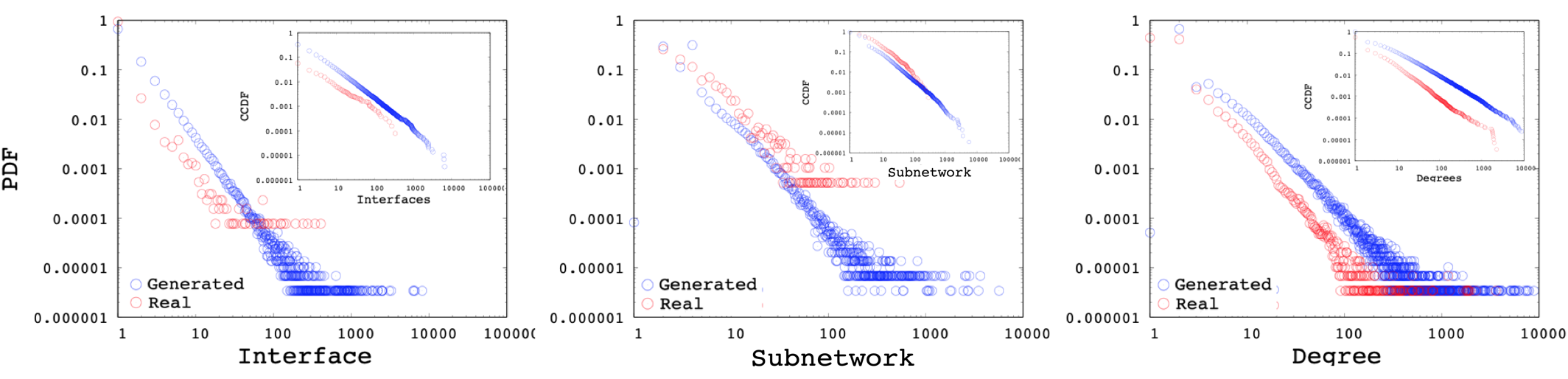

Supplement: S1 File — (ZIP) [file pone.0240100.s001.zip › SubNetG_ revision/figures/8928.png]

**a**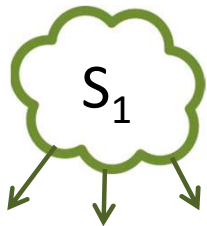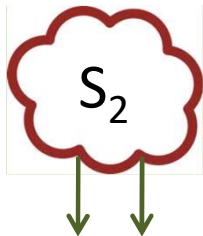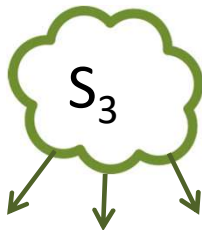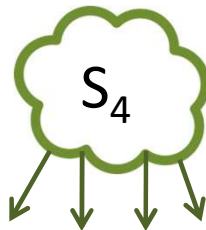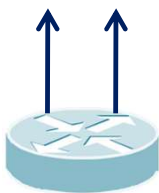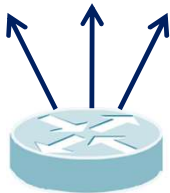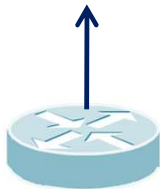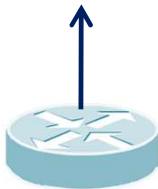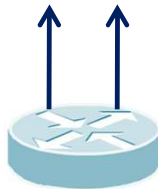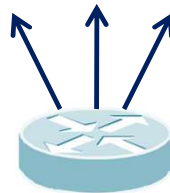 $R_1$  $R_2$  $R_3$  $R_4$  $R_5$  $R_6$

Supplement: S1 File — (ZIP) [file pone.0240100.s001.zip › SubNetG_ revision/figures/Alg2-steps1.pdf]

**b**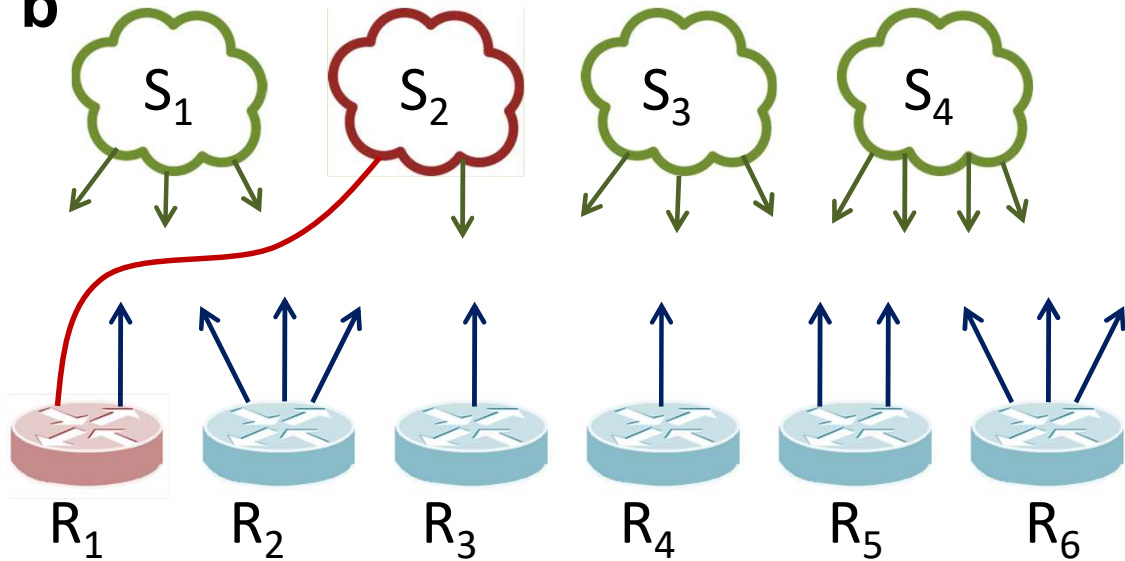

Supplement: S1 File — (ZIP) [file pone.0240100.s001.zip › SubNetG_ revision/figures/Alg2-steps2.pdf]

**c**

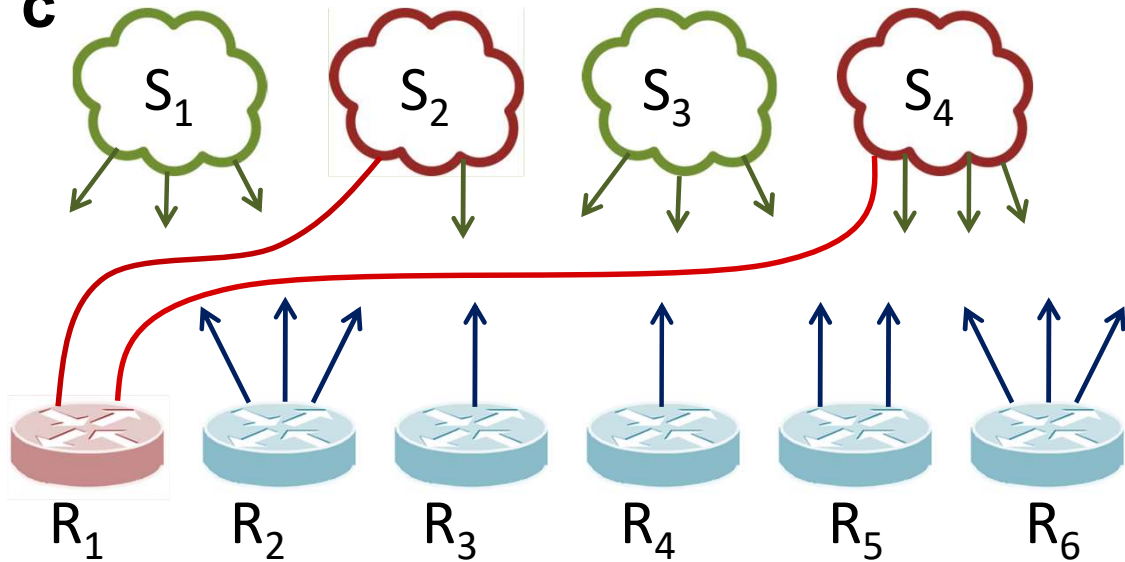

Supplement: S1 File — (ZIP) [file pone.0240100.s001.zip › SubNetG_ revision/figures/Alg2-steps3.pdf]

**d**

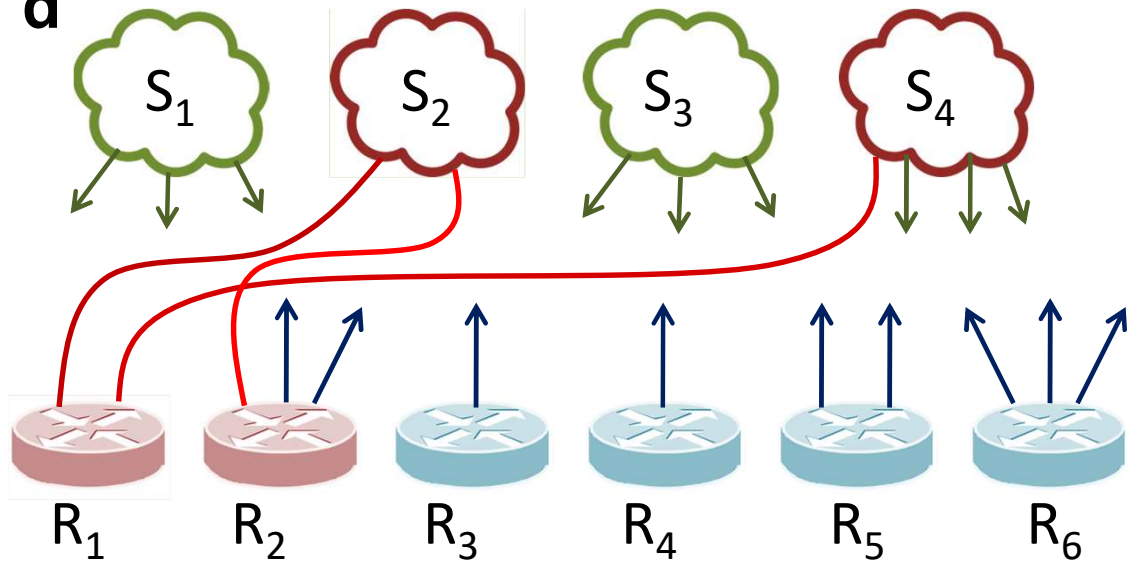

Supplement: S1 File — (ZIP) [file pone.0240100.s001.zip › SubNetG_ revision/figures/Alg2-steps4.pdf]

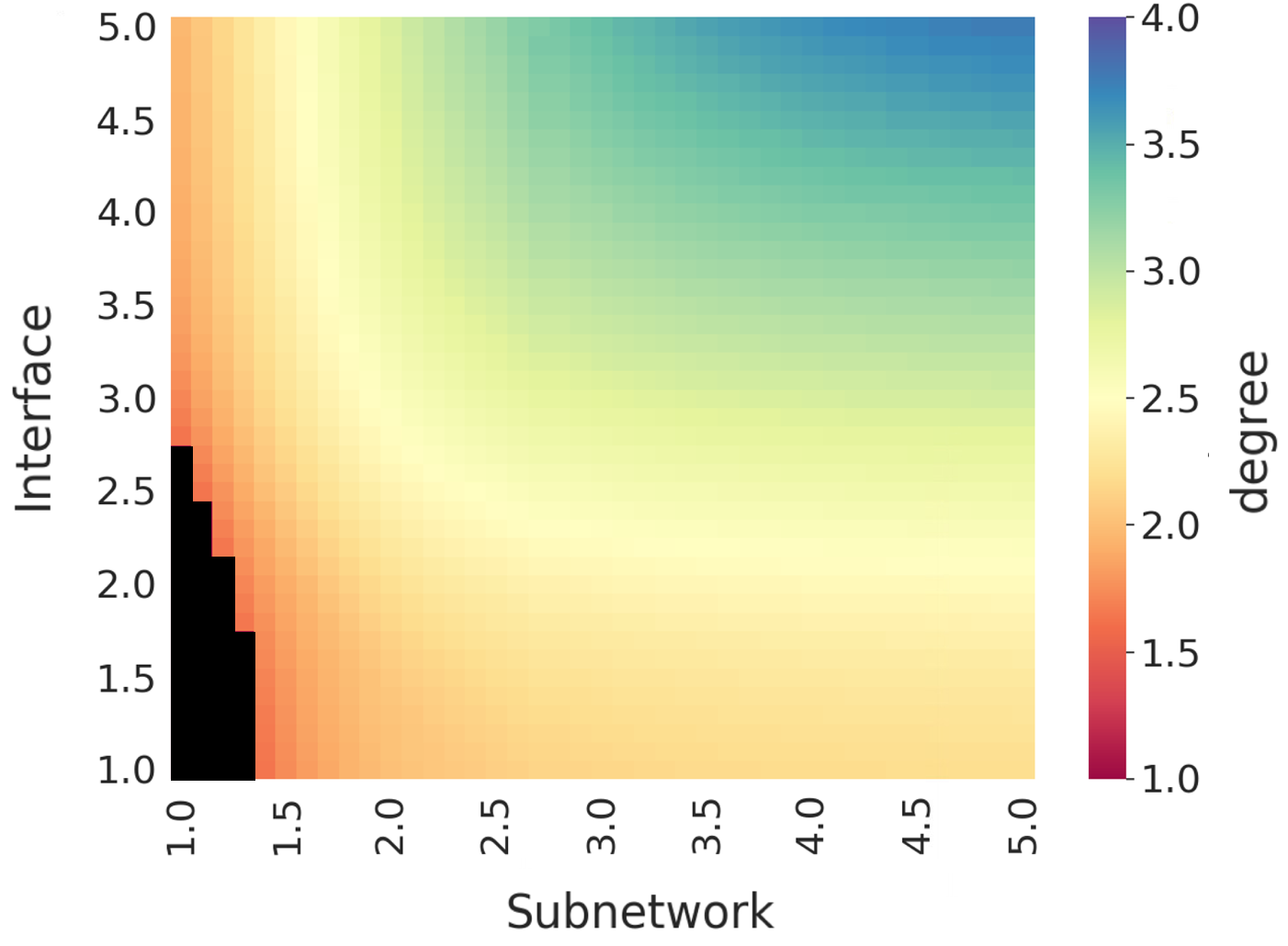

Supplement: S1 File — (ZIP) [file pone.0240100.s001.zip › SubNetG_ revision/figures/alphaCorrelation.png]

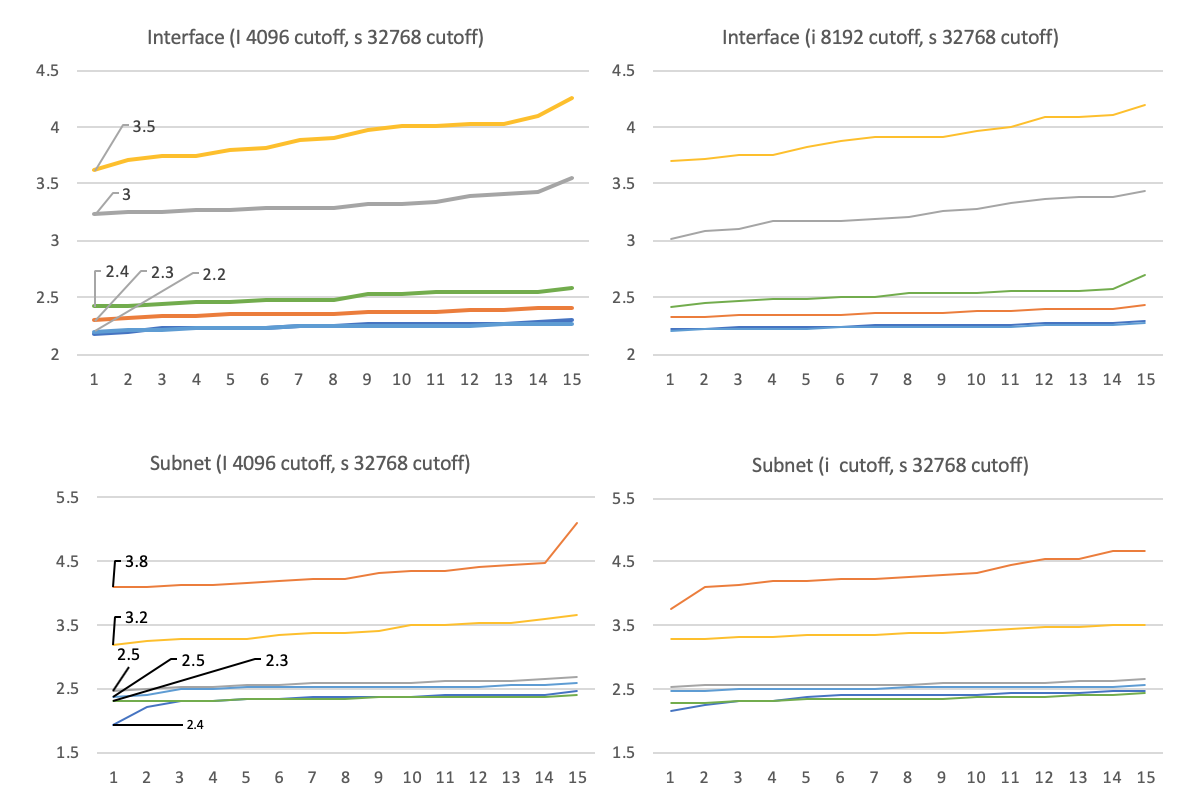

Supplement: S1 File — (ZIP) [file pone.0240100.s001.zip › SubNetG_ revision/figures/AlphaValues.png]

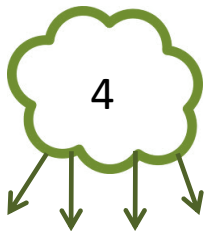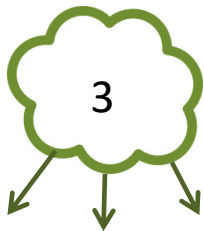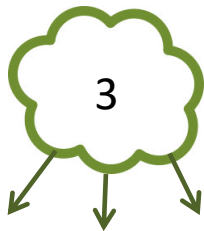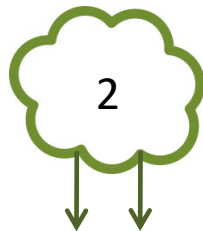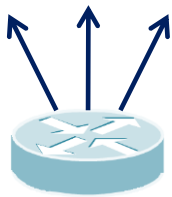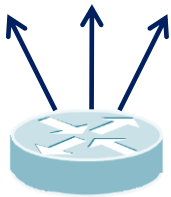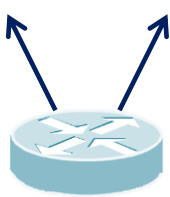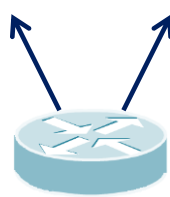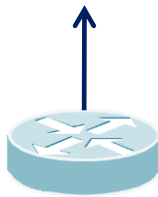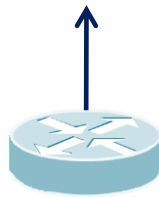

3

3

2

2

1

1

Supplement: S1 File — (ZIP) [file pone.0240100.s001.zip › SubNetG_ revision/figures/bipartite.pdf]

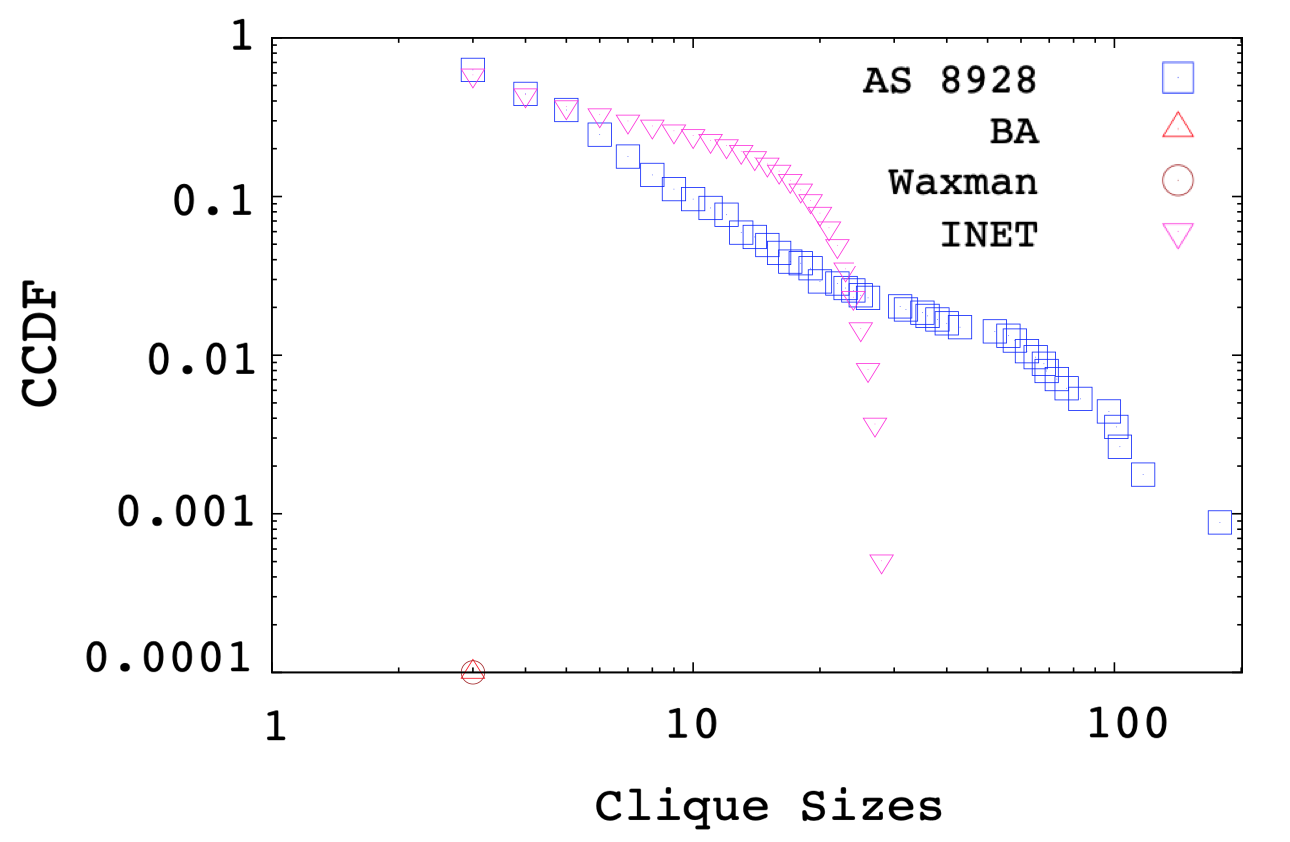

Supplement: S1 File — (ZIP) [file pone.0240100.s001.zip › SubNetG_ revision/figures/cliques/CliqCCDF8928.png]

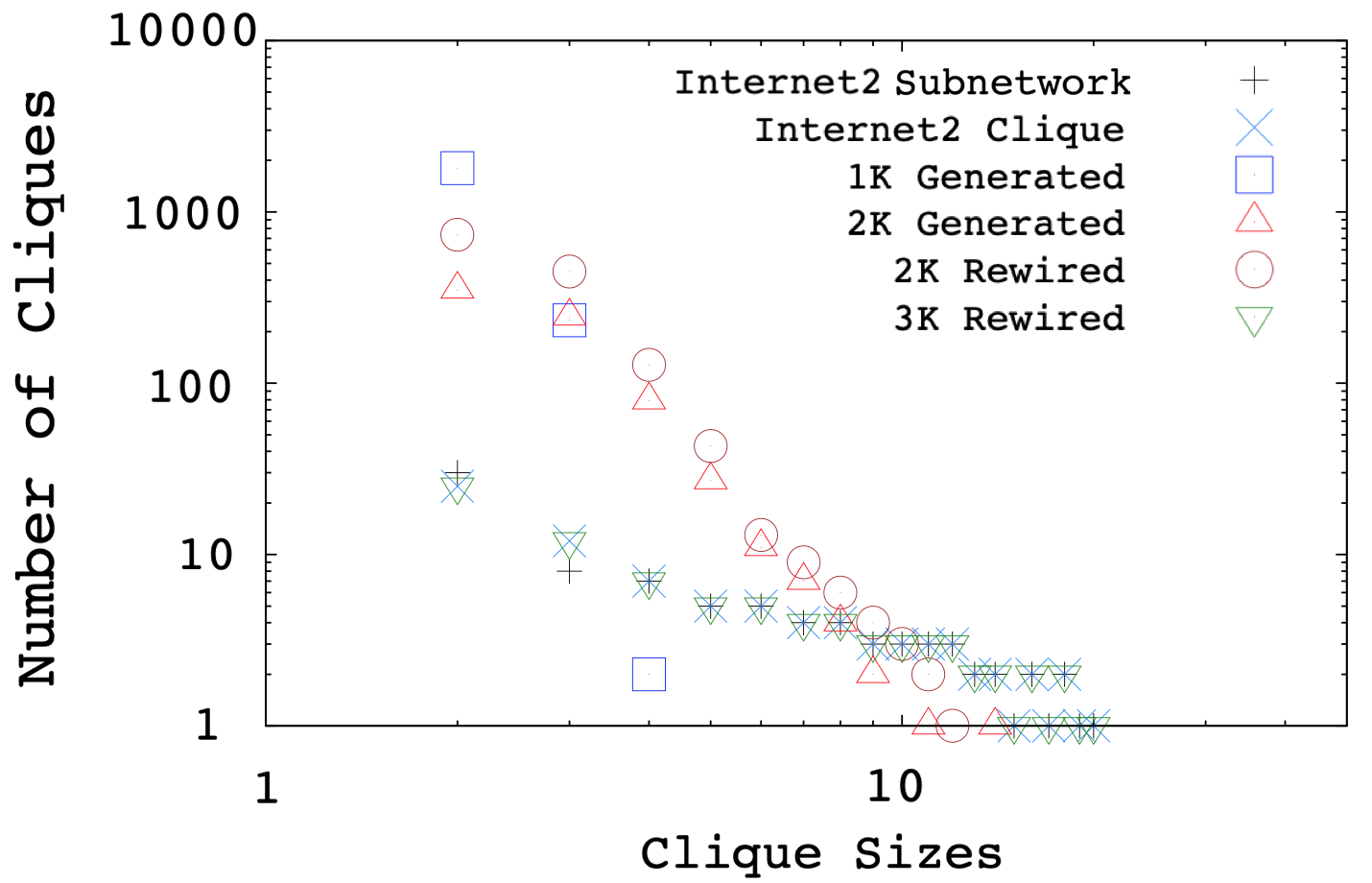

Supplement: S1 File — (ZIP) [file pone.0240100.s001.zip › SubNetG_ revision/figures/cliques/Internet2.png]

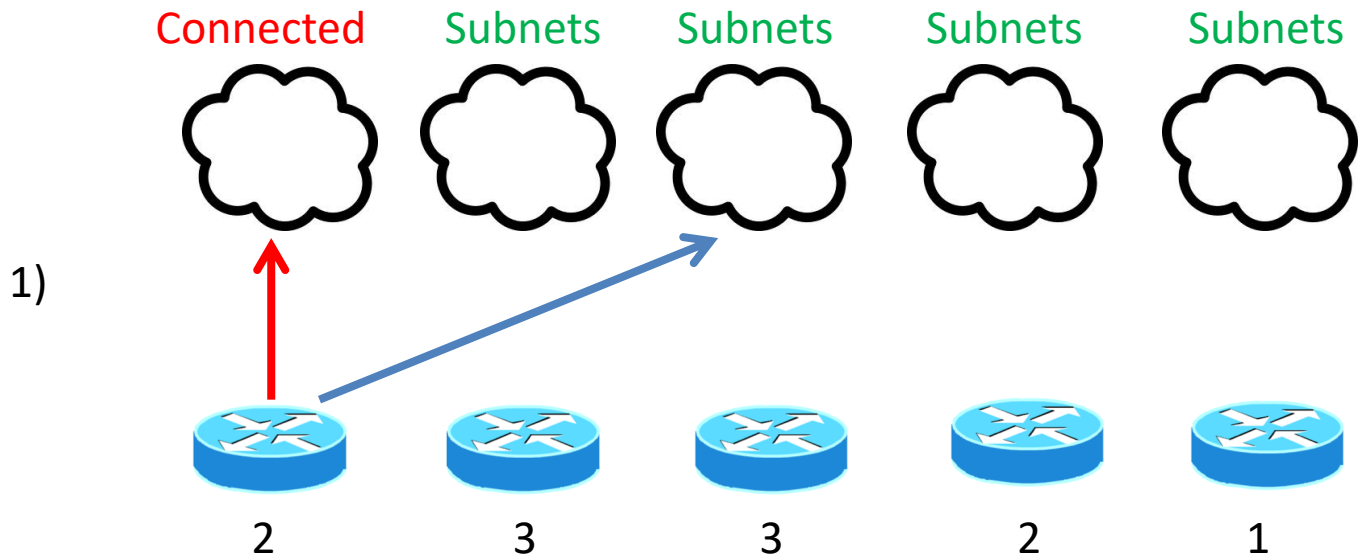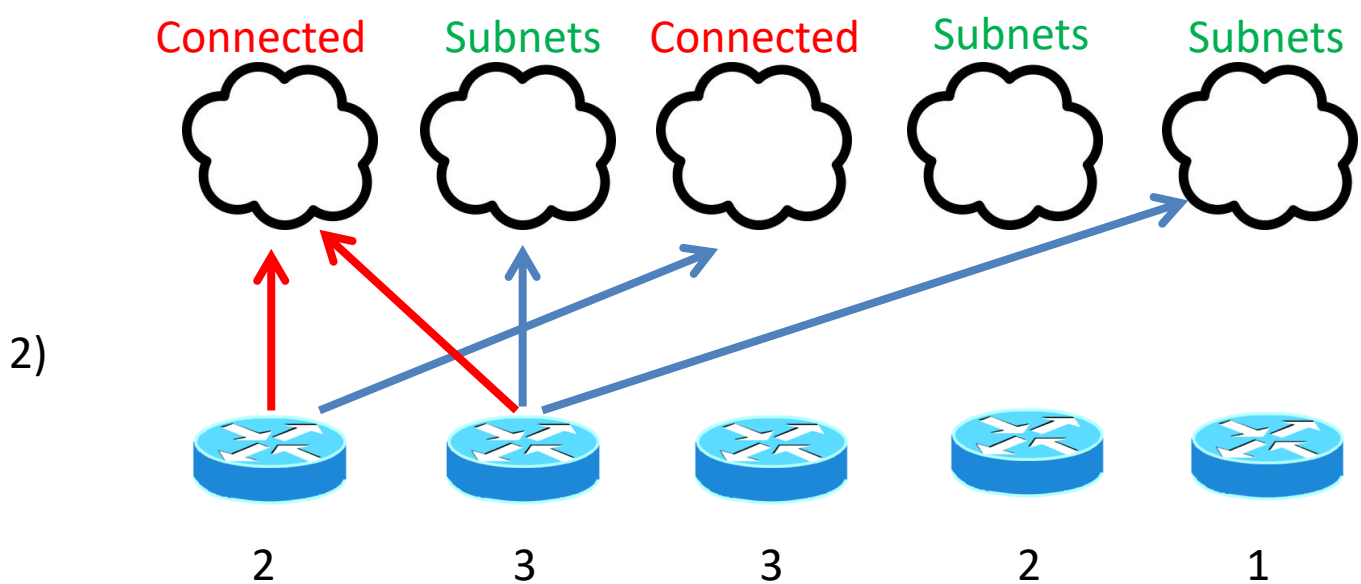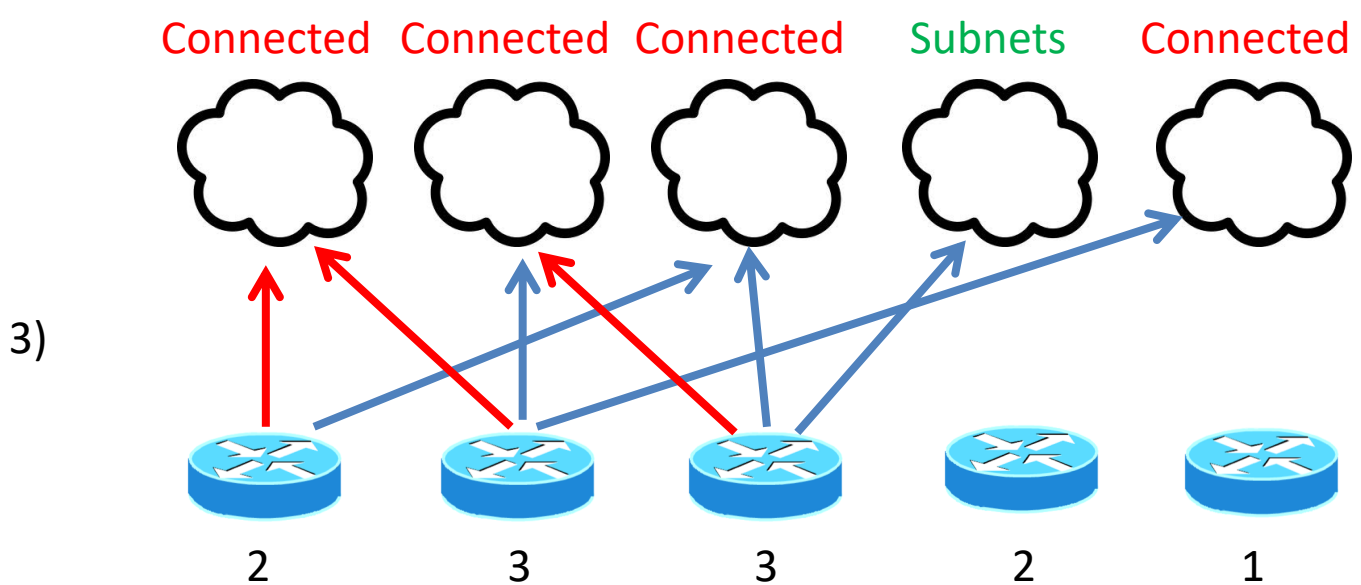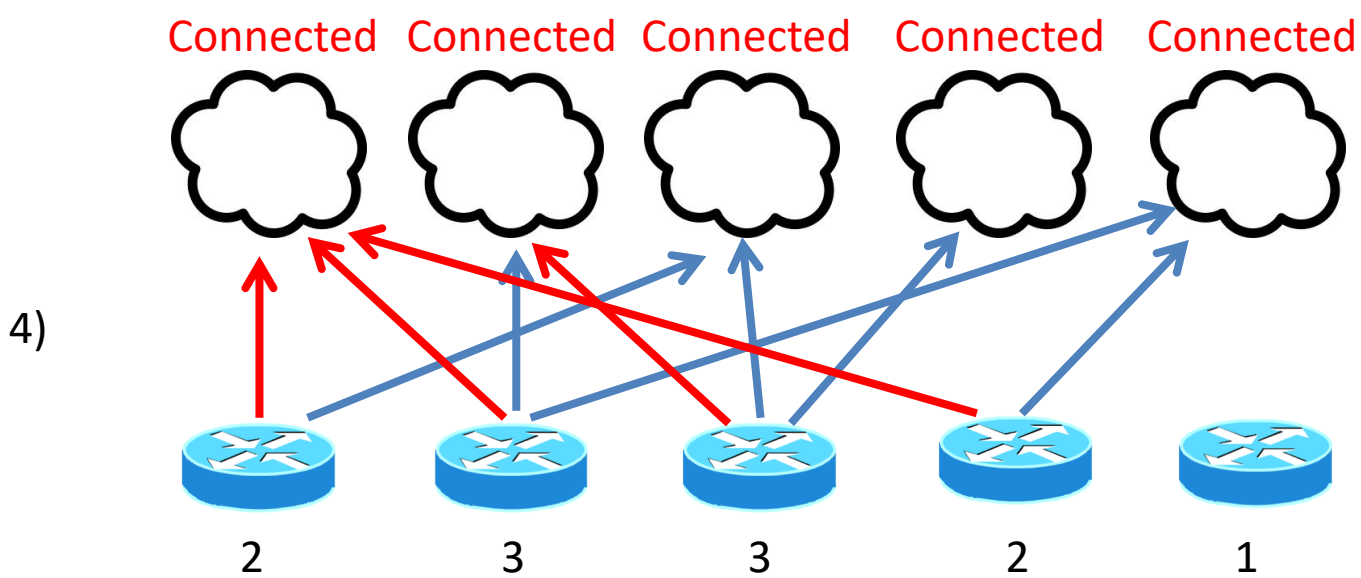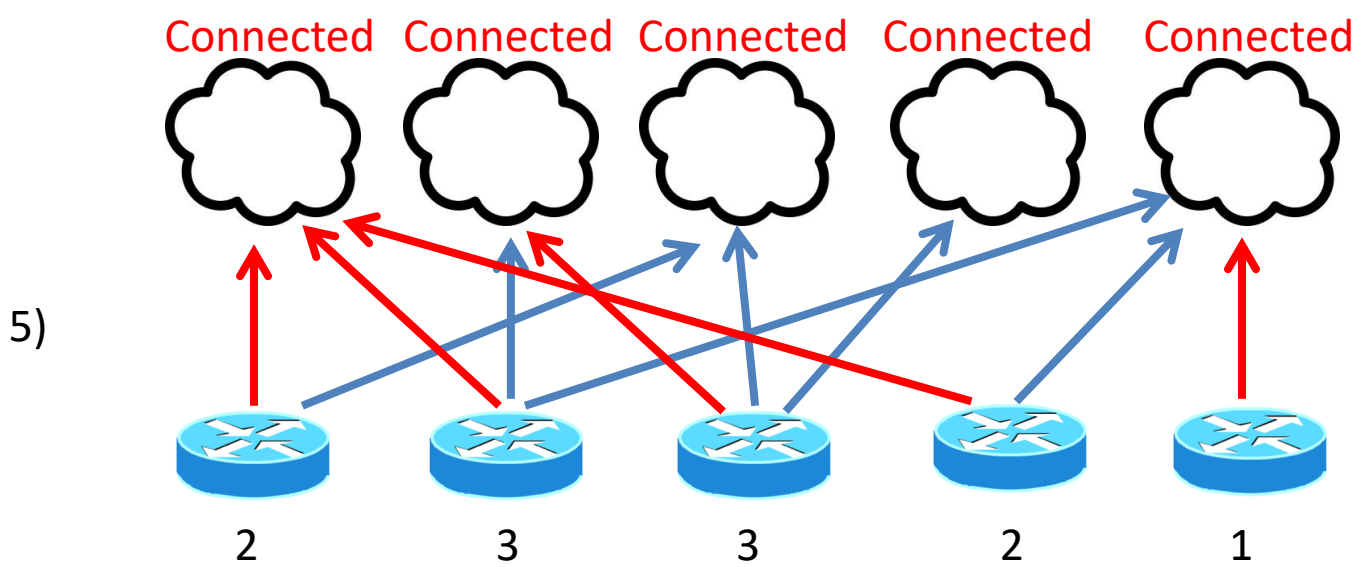

Supplement: S1 File — (ZIP) [file pone.0240100.s001.zip › SubNetG_ revision/figures/connectivity_toy_example.pdf]

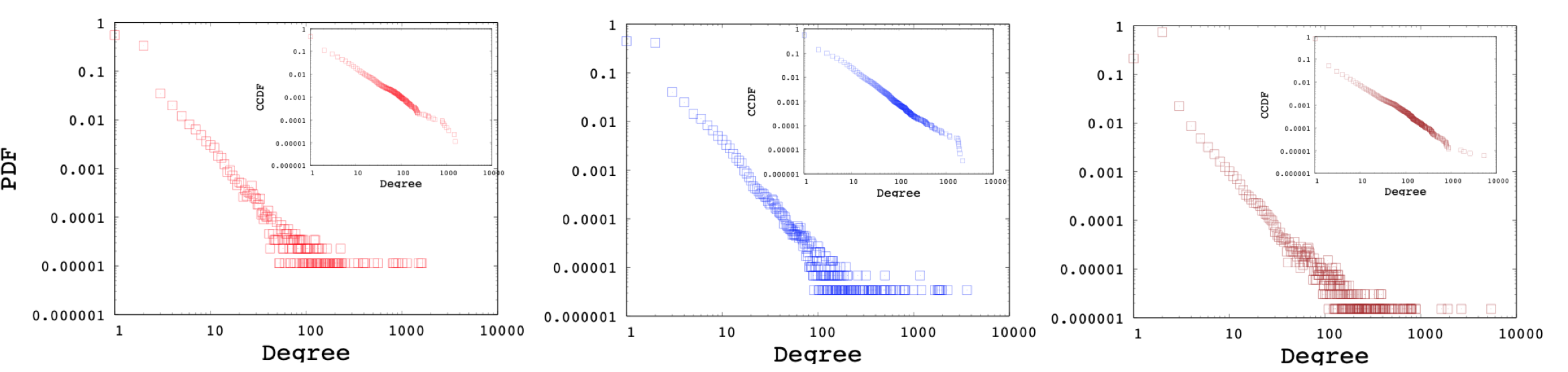

Supplement: S1 File — (ZIP) [file pone.0240100.s001.zip › SubNetG_ revision/figures/degree.png]

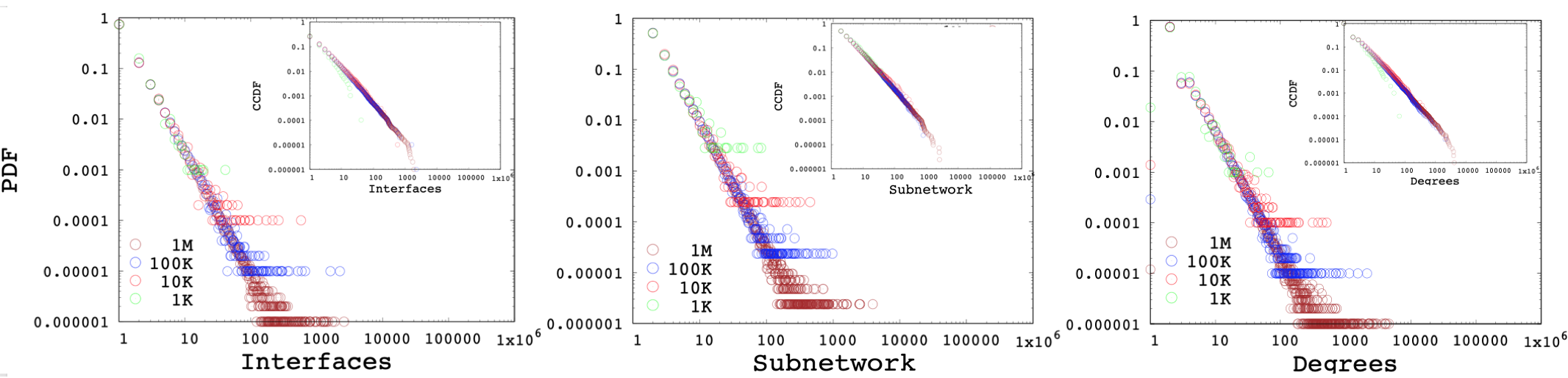

Supplement: S1 File — (ZIP) [file pone.0240100.s001.zip › SubNetG_ revision/figures/generated1K10K100K1M_2.png]

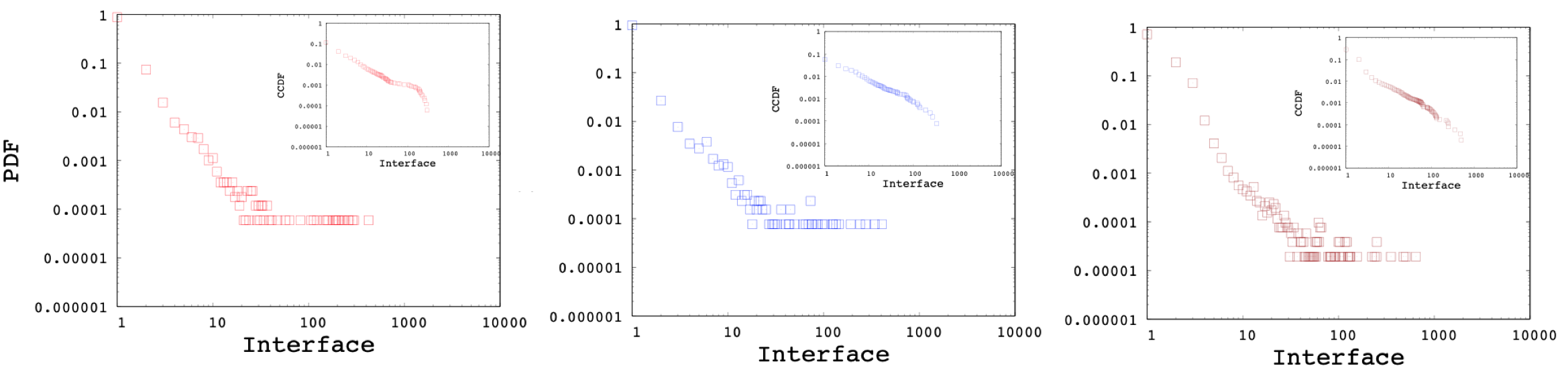

Supplement: S1 File — (ZIP) [file pone.0240100.s001.zip › SubNetG_ revision/figures/interface.png]

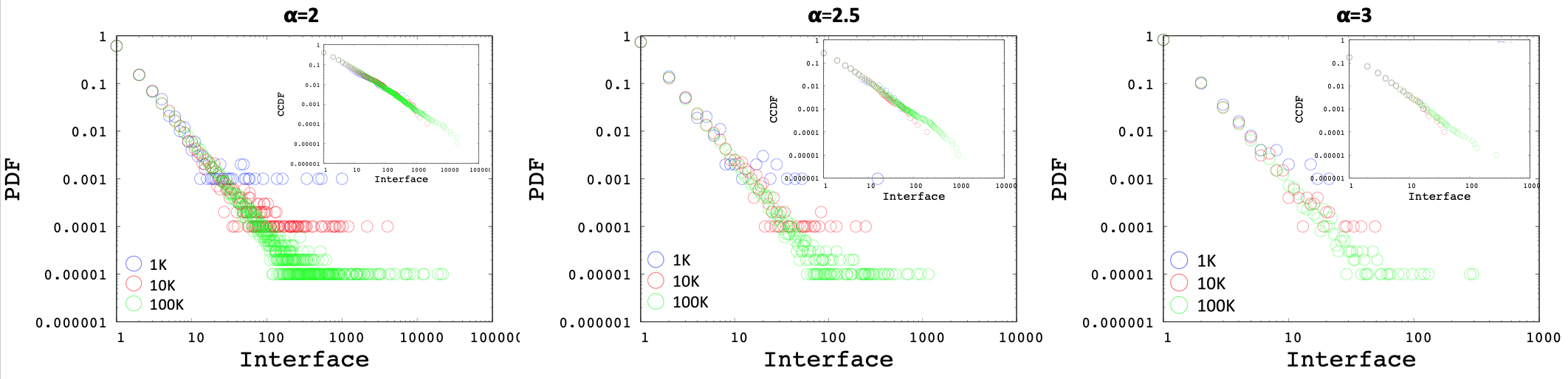

Supplement: S1 File — (ZIP) [file pone.0240100.s001.zip › SubNetG_ revision/figures/InterfacesGenerated.png]

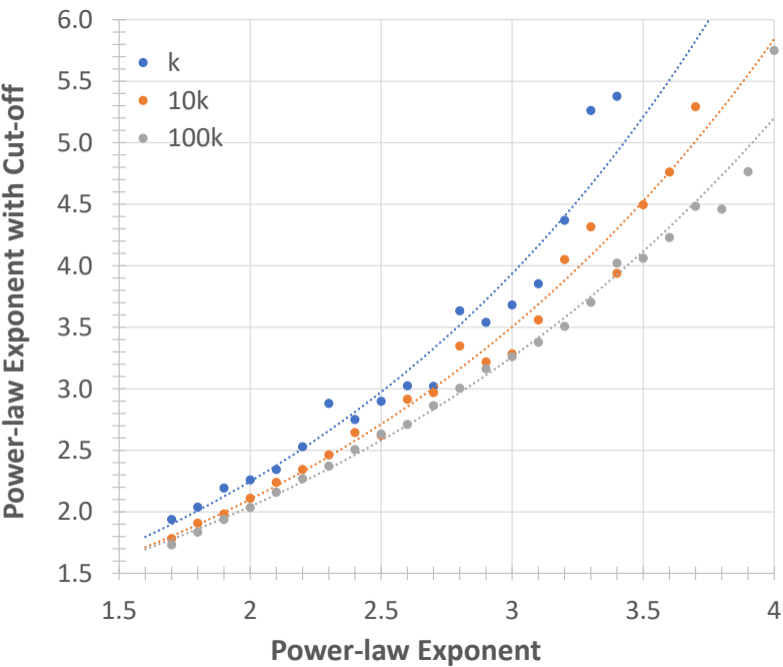

Supplement: S1 File — (ZIP) [file pone.0240100.s001.zip › SubNetG_ revision/figures/medians.pdf]

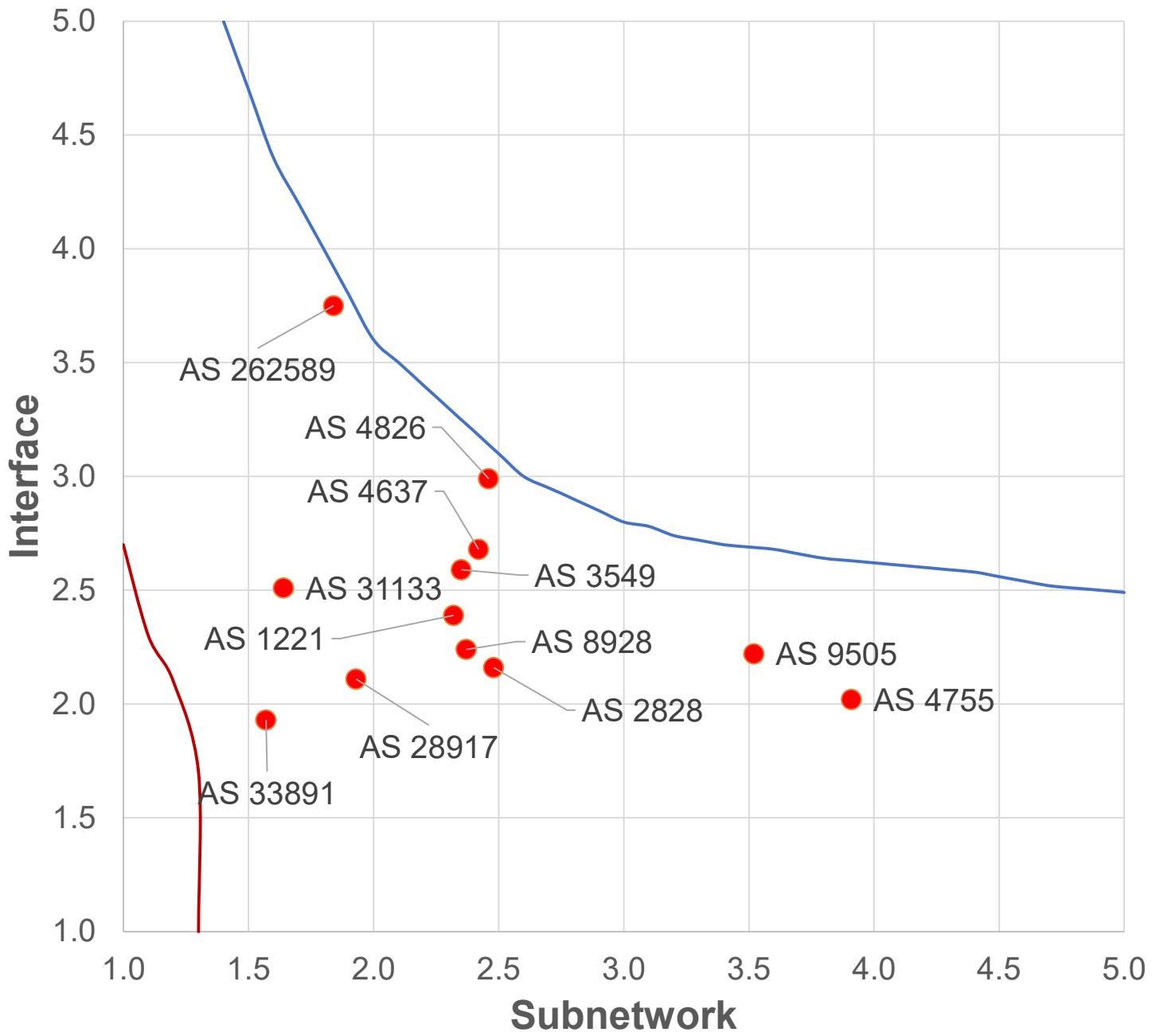

Supplement: S1 File — (ZIP) [file pone.0240100.s001.zip › SubNetG_ revision/figures/power-law-values.pdf]

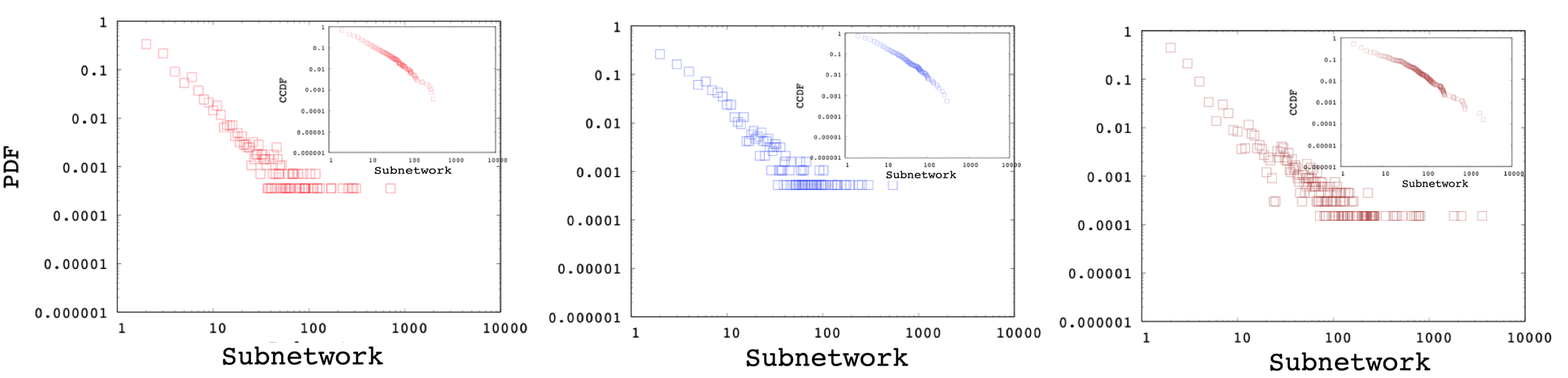

Supplement: S1 File — (ZIP) [file pone.0240100.s001.zip › SubNetG_ revision/figures/subnet.png]

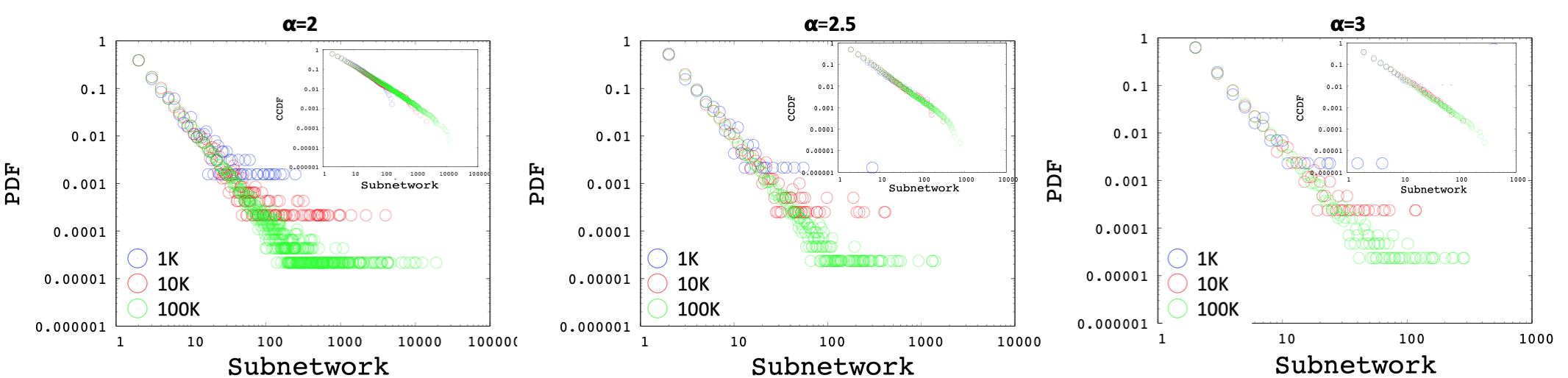

Supplement: S1 File — (ZIP) [file pone.0240100.s001.zip › SubNetG_ revision/figures/SubnetsGenerated.png]

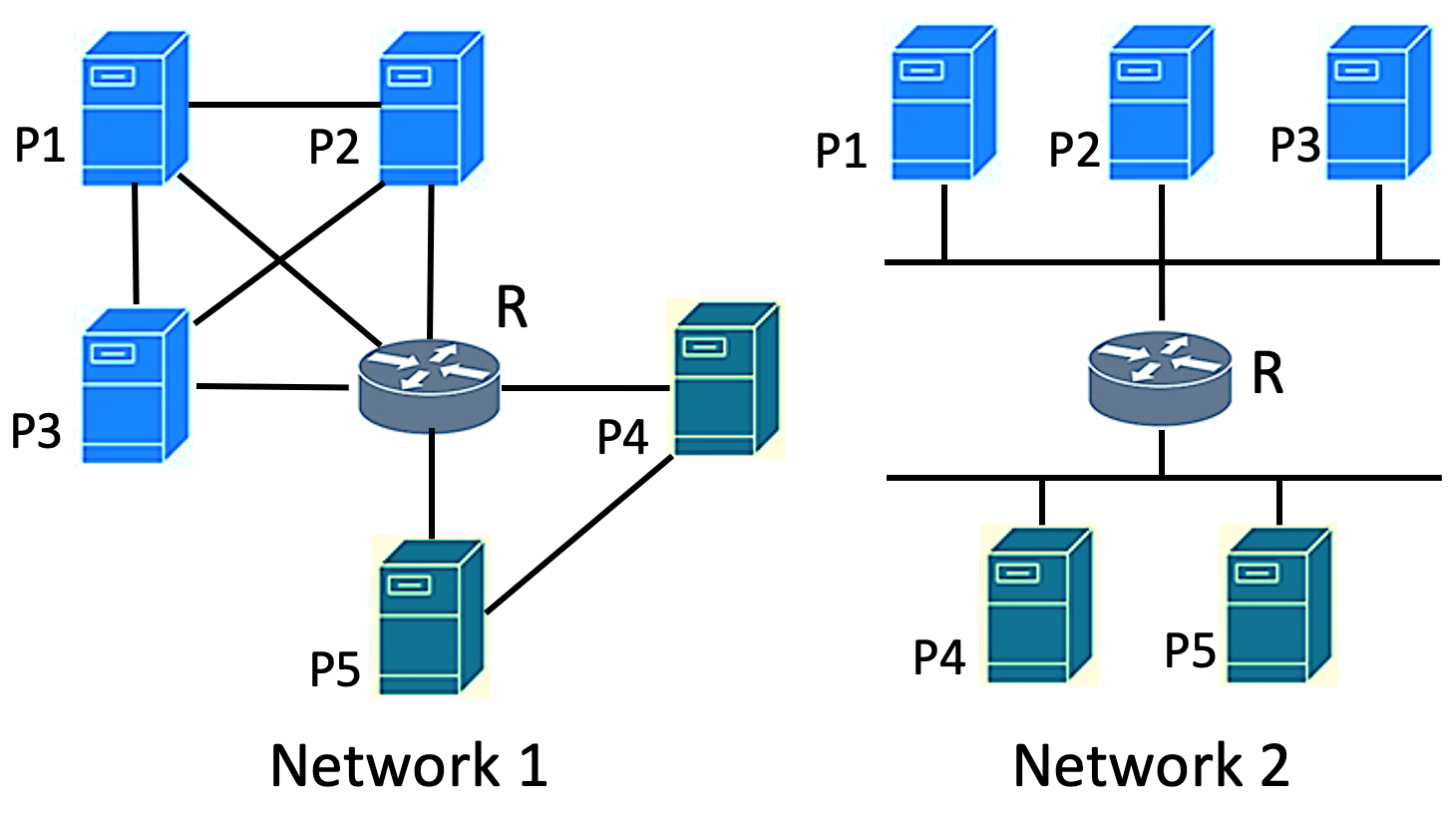

Supplement: S1 File — (ZIP) [file pone.0240100.s001.zip › SubNetG_ revision/figures/topology_1.png]

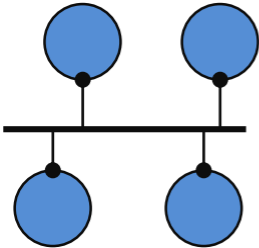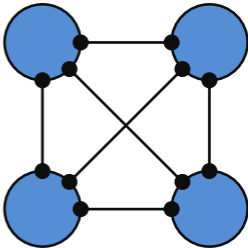

Supplement: S1 File — (ZIP) [file pone.0240100.s001.zip › SubNetG_ revision/figures/topology_2.pdf]

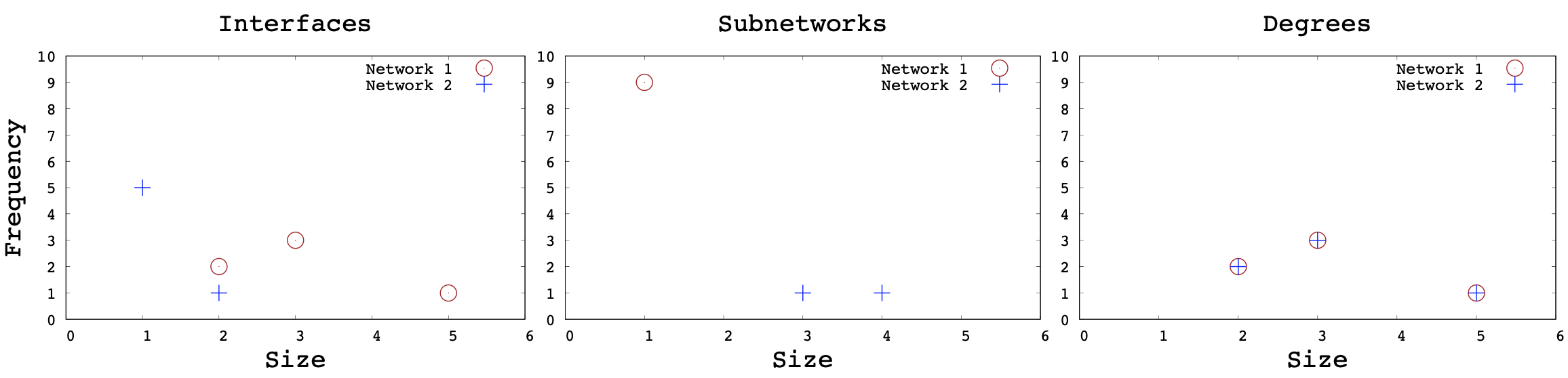

Supplement: S1 File — (ZIP) [file pone.0240100.s001.zip › SubNetG_ revision/figures/topology_dist.png]
